# Hereditable variants of classical protein tyrosine phosphatase genes: Will they prove innocent or guilty?

**DOI:** 10.3389/fcell.2022.1051311

**Published:** 2023-01-23

**Authors:** Wiljan J. A. J. Hendriks, Remco T. P. van Cruchten, Rafael Pulido

**Affiliations:** ^1^ Department of Cell Biology, Radboud University Medical Centre, Nijmegen, The Netherlands; ^2^ Department of Pathology, Radboud University Medical Centre, Nijmegen, The Netherlands; ^3^ Biomarkers in Cancer Unit, Biocruces Bizkaia Health Research Institute, Barakaldo, Spain; ^4^ Ikerbasque, Basque Foundation for Science, Bilbao, Spain

**Keywords:** disease susceptibility, gene mutation, hereditable disease, phosphotyrosine, posttranslational modification, signal transduction, single nucleotide polymorphism

## Abstract

Protein tyrosine phosphatases, together with protein tyrosine kinases, control many molecular signaling steps that control life at cellular and organismal levels. Impairing alterations in the genes encoding the involved proteins is expected to profoundly affect the quality of life—if compatible with life at all. Here, we review the current knowledge on the effects of germline variants that have been reported for genes encoding a subset of the protein tyrosine phosphatase superfamily; that of the thirty seven classical members. The conclusion must be that the newest genome research tools produced an avalanche of data that suggest ‘guilt by association’ for individual genes to specific disorders. Future research should face the challenge to investigate these accusations thoroughly and convincingly, to reach a mature genotype-phenotype map for this intriguing protein family.

## 1 Introduction

Life, be it from a cellular, organismal or population view point, requires abilities to respond quickly and effectively to dynamic changes imposed by the environment. At a molecular level, the smallest unit of life—the cell—has developed strategies that make use of an extensive collection of fast and reversible modifications to its biomolecules. Especially for processes that impact on survival, migration, growth, proliferation and differentiation eukaryotic cells exploit reversible phosphorylation of specific amino acid residues in relevant target proteins, with the aim to quickly alter their activities, stabilities, interactions and/or subcellular localizations (Hunter, 1995). These reversible post-translational modifications are the net result of the opposing activities of two sets of cellular enzymes: protein kinases and protein phosphatases. The majority of protein (de) phosphorylation events addresses serine and/or threonine residues. However, in multicellular organisms the specific (de) phosphorylation of tyrosine residues became more and more the method of choice for regulation of cell division and cell diversity (Lim and Pawson, 2010). In line, the molecular causes in acquired or hereditary diseases of growth and development are regularly mapped on phosphotyrosine-mediated signaling routes (e.g., (Gelb and Tartaglia, 2006; Vogelstein et al., 2013)). Consequently, a considerable number of protein tyrosine kinase genes are now documented as proto-oncogenes or are linked to developmental disorders. Encoding their enzymatic counterparts, the protein tyrosine phosphatase (PTP) genes were therefore initially viewed as housekeeping genes with tumor suppressor potential and perhaps with impact on differentiation processes as well (Tonks, 2013). Now, 34 years after the first PTP enzyme was isolated and characterized (Tonks et al., 1988) and with more than twenty thousand “tyrosine phosphatase”-containing articles in PubMed, we realize that life is much more complex.

**FIGURE 1:**
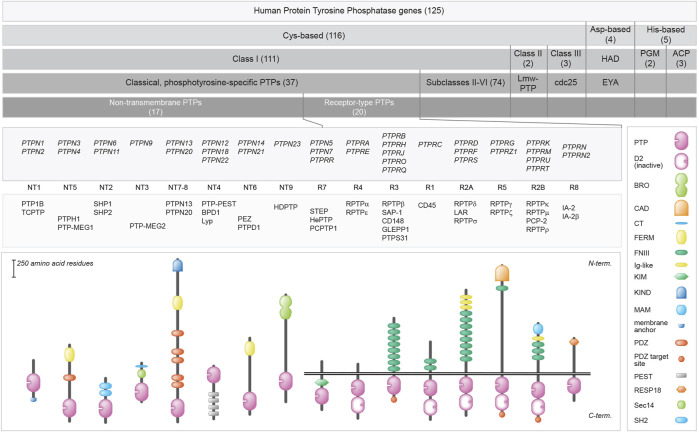
Classification of protein tyrosine phosphatase genes, and protein domain structure for the 37 classical PTPs. Upper 5 bars summarize the build-up of (sub) classes within the family. The number of genes within the (sub) classes are indicated in between brackets. Below, the gene (italics), subtype codes, and protein names of the classical PTPs, as well as their schematic structures, are given. The horizontal twin black lines represent the cell membrane. Protein domain representations are explained on the right. BRO, yeast Bro1 homologous domain; CAD, carbonic anhydrase domain; CT, CRAL-TRIO N-terminal homology domain; D2, inactive PTP domain; FERM, 4.1 protein-ezrin-radixin-moesin; FNIII, Fibronectin type three; Ig-like, Immunoglobulin-like; KIM, kinase interaction motif; KIND, kinase non-catalytic N-lobe; MAM, meprin, A-5 neuropilin, and RPTPμ; PDZ, postsynaptic density-95/discs large/ZO1 homology; PEST, proline-glutamic acid-serine-threonine-rich area; Pro-rich, proline-rich area; PTP, catalytic domain; RESP18, regulated endocrine-specific protein-18 homology domain; Sec14, Sec14 homology; SH2, Src Homology 2. Classification is according to Alonso and Pulido (Alonso et al., 2016; Alonso and Pulido, 2016). Subtype codes come from Andersen *et al.* (Andersen et al., 2004).

One hundred and twenty five genes in the human genome encode PTP family members, and a closer look reveals that multiple chemical mechanisms are exploited by subfamilies to get the job—dephosphorylating phosphotyrosine-containing or alternative substrates—done (Hunter, 1995; Lim and Pawson, 2010; Tonks, 2013; Alonso and Pulido, 2016). The largest group encodes proteins that use cysteine in their catalytic site as the essential residue for a two-step enzymatic mechanism, but other subgroups rely on aspartate or on histidine. The first enzymatic step is an attack by the active site Cys residue on the phosphorus atom in the substrate, resulting in the formation of a covalent thiophosphate enzyme intermediate. The second, rate-limiting step requires a water molecule to attack the phosphorus atom, effectively separating inorganic phosphate and the PTP. The cysteine-based group harbors a cluster of so-called ‘classical PTPs’ that were thought to be phosphotyrosine-specific, whereas the remaining largest part demonstrated more broad specificities and could also dephosphorylate phosphoserines and phosphothreonines, and occasionally also phospholipids or phosphorylated carbohydrates. Traditionally, the classical PTPs have been further split into two groups; non-transmembrane PTPs and receptor-type PTPs, although for most the potential ligands and their effect upon binding still need to be discovered (Mohebiany et al., 2013). As will become obvious during the following, the discovery of many different isoforms encoded by classical PTP genes has blurred this clear separation. Furthermore, the type of enzymatic activities displayed by some of the classical PTPs also goes far beyond phosphotyrosines. Figure 1 provides an overview of the superfamily of PTPs, with representative protein domain composition for the various subgroups of classical PTPs.

The technical possibilities to investigate disease processes at the genomic level have exploded over the past decades (reviewed in (de Bruijn et al., 2021)), and consequently huge collections of DNA sequence variabilities between individuals have been gathered. Whole exome sequencing (WES) and increasingly whole genome sequencing (WGS) approaches have yielded numerous allele variants for genes that differ at specific nucleotide positions (SNPs, single nucleotide polymorphisms) or represent structural variants (i.e. comprise insertions or deletions of multiple nucleotides in a row). Furthermore, gene expression levels may be influenced by copy number variations (CNVs) as well. And whereas in the 90s disease gene identification relied on family-based linkage studies using a collection of genomic markers (de Bruijn et al., 2021), one can nowadays turn to genome-wide approaches with samples from unrelated individuals in genome-wide association studies (GWAS) or transcriptome-wide association studies (Gamazon et al., 2015; Visscher et al., 2017). Tailored databases open up rich resources for consultation by the research community. The gnomAD database, for example, contains exome and genome data, which can be used to assess the population frequency of SNPs and other variants. OMIM (Online Mendelian Inheritance in Man) provides a list of human genes and their associated phenotypes. The ClinVar database contains reports of the clinical significance, associated phenotypes, and supporting evidence for genetic variants, and the Gene Curation Coalition (GenCC) database provides information about the validity of gene-disease relationships.

With the growing list of genomic sequences, it now becomes the challenge to link the various polymorphisms to eventual phenotypic consequences. Variant interpretation is of course helped by the online resources mentioned above. Here, we present a comprehensive overview of currently documented sequence variants in the classical subset of PTP genes, and discuss existing or suspected links with hereditary human pathologies or disease susceptibilities. This not only sheds light on the degrees of freedom within the structures of the encoded, highly conserved proteins but will also facilitate PTP gene sequence interpretations in future samples.

## 2 Documented genetic variability

### 2.1 Methodology

We monitored for publications that appeared in PubMed over the years following our comprehensive 2013 review on PTP genes and their disease associations (Hendriks and Pulido, 2013). For some missense mutations we ran HOPE analyses (Venselaar et al., 2010) to gather structural information about the consequences at the protein level. Next to this, the current list of thirty seven classical PTP genes, as defined in (Alonso et al., 2004), was used and corresponding chromosomal coordinates were extracted based on gene IDs in *via entrez_search* in the rentrez R-Package (Winter 2017; Team, 2022). Missense, nonsense, and frameshift SNPs that mapped in these regions were collected from *dbSNP 151 common via* https://genome.ucsc.edu/cgi-bin/hgTables, yielding 118 common SNPs that were not identified in the literature search. SNP attributes were queried in the ensembl_mart_snp database (Cunningham et al., 2022) *via* BioMart (Durinck et al., 2005). Furthermore, OMIM and ClinVar databases were inspected using gene names as queries, and relevant and well-documented entries were selected *via* manual curation. The findings (summarized in Supplementary Table S1) will be discussed below, in an order that matches the subtyping used in Figure 1. The list of variants in respective databases is much larger, e.g., for *PTPN11, PTPN23 and PTPRC*. For a comprehensive list the reader is therefore referred to these online resources. We also mined the recent Gene Curation Coalition database (DiStefano et al., 2022), which yielded findings for nine of the 37 classical PTP genes (Table 1).

**TABLE 1:** Curated data for PTP genes in GenCC.

*Gene*	*Disease(s)*	*Evidence* *^a^*
*PTPN3*	Schizophrenia	Unknown
*PTPN11*	Noonan syndrome, Noonan syndrome with multiple lentigines	Definitive
	Metachondromatosis	Strong
	Costello syndrome, cardiofaciocutaneous syndrome	Disputed
*PTPN14*	Lymphedema-posterior choanal atresia syndrome	Strong
*PTPN22*	IDDM 1, rheumatoid arthritis	Limited
*PTPRC*	Severe combined immunodeficiency, autosomal recessive, T cell-negative, B cell-positive, NK cell-positive	Strong
*PTPRF*	Breasts and/or nipples, aplasia or hypoplasia	Strong
*PTPRJ*	Hereditary nonpolyposis colon cancer	Limited
	Colorectal cancer	Unknown
*PTPRO*	Familial idiopathic steroid-resistant nephrotic syndrome	Supportive
	Nephrotic syndrome, type 6	Limited
*PTPRQ*	Hearing loss, autosomal recessive	Definitive
	Autosomal recessive nonsyndromic hearing loss 84A	Strong
	Hearing loss, autosomal dominant 73	Moderate
	Autosomal dominant nonsyndromic hearing loss	Supportive

^a^ Evidence in GenCC (DiStefano et al., 2022) is rated either as Definitive (repeatedly demonstrated in both the research and clinical diagnostic settings, and upheld over time), Strong (repeatedly and independently demonstrated in humans and no conflicting evidence), Moderate (moderate evidence in humans, no contradictory evidence), Limited (little human evidence to support a causal role, but not all has been refuted), Unknown (no disease claim in any organism has been made).

### 2.2 *PTPN1* and *PTPN2*

The human genes *PTPN1* and *PTPN2* encode the two members of the NT1 subtype of classical PTPs, PTP1B and TCPTP. They are amongst the smallest PTP proteins, essentially consisting of the ∼250 amino acids long catalytic PTP domain and a C-terminal stretch that determines their subcellular localization. Both are ubiquitously expressed and despite their similarity they exert distinct functions as became evident from mouse knockout studies and the analyses of leukemic and lymphoid tumor samples (Pike and Tremblay, 2016). The data implicate that both are negative regulators of the JAK-STAT pathway and therefore PTP1B or TCPTP loss will contribute to leukemogenesis or lymphomagenesis. Furthermore, a strong link between PTP1B and glucose metabolism has been established (Feldhammer et al., 2013). Consequently, several sequence variants have been identified in recent years that can be linked to pathologies in human (Supplementary Table S1).

*PTPN1* alleles harboring SNP rs16989673, the 1484insG sequence variant in the 3’ untranslated region of the human PTP1B-encoding mRNA, produce transcripts that are more stable. This results in higher levels of the PTP1B enzyme and increases the risk to develop insulin resistance (Di Paola et al., 2002). Likewise, the PTP1B 981^−ΔΔCT^ genotype was shown to reduce the risk to develop noninsulin-dependent diabetes mellitus (Mok et al., 2002) and the p.P387L missense variant did the reverse. Also IVS6+82G-A heterozygotes have a higher risk for type 2 diabetes (Ukkola et al., 2005). Several SNPs, including IVS6+82G-A, that are found in Japanese and Chinese populations have been linked to a metabolic syndrome with affected plasma lipid levels, obesity and hypertension (Olivier et al., 2004). Notably, their findings for the IVS6+82G-A SNP point to a genetic interaction between PTPN1 and the leptin receptor gene, corroborating earlier mouse work (Cheng et al., 2002; Zabolotny et al., 2002).

For PTPN2 some more SNPs with pathological connotations have been reported (Supplementary Table S1), and over the years these have built a strong portfolio for the gene’s impact on inflammatory processes (Wellcome Trust Case Control Consortium, 2007; Long et al., 2011; Pike and Tremblay, 2016). A recent meta-analysis (Barrett et al., 2008) corroborated the link between PTPN2 variants and inflammatory bowel diseases, notably Crohn disease (CD). For some of these SNPs, e.g., rs78174797 that describes the missense mutation p.T171K in TCPTP, it is rather difficult to predict what the effect at the protein level will be. Using HOPE (Venselaar et al., 2010) one can predict that the substitution takes place at the surface of the phosphatase domain but whether this impacts on the enzyme’s interaction possibilities including substrate specificity, remains to be established. The situation is much more clear for the recently identified p.C216G variant (Parlato et al., 2020) because the amino acid change here involves the essential catalytic site cysteine (Nian et al., 2022). This renders the mutant protein enzymatically dead and consequently potentially substrate-trapping because catalysis is blocked but substrate affinity is maintained (Blanchetot et al., 2005). TCPTP haploinsufficiency results in intestinal autoimmunity and, since enterocolitis is also caused by STAT3 gain-of-function mutations, it comes as no surprise that JAK-STAT hyperactivity in immune cells represents the downstream effect. Moreover, a rare variant (rs80191532; *p* = 9.3 × 10^–7^) discovered in a family as part of a cohort-wide WGS approach implicated *PTPN2* in primary immunodeficiencies (Thaventhiran et al., 2020), which are known by frequent infections that can be life-threatening. Authors found evidence for genetic interplay between variants in the *PTPN2* and *SOCS1* gene regions. SOCS1 (Suppressor of cytokine signaling 1) is part of negative-feedback signaling pathways downstream of cytokine receptors, including the interferon gamma receptor, that downregulates STAT-mediated signals, and a protein truncating mutation in SOCS1 was causally related to primary immunodeficiency (Thaventhiran et al., 2020). These studies lead to a model where the severity of the disease inversely correlates with remaining TCPTP activity, with haploinsufficiency causing autoimmunity and any further decrease in activity as a result of more common variants in the second PTPN2 allele leading to immunodeficiency.

Since the chronic immune conditions to which *PTPN2* is linked - Crohn’s disease (CD), ulcerative colitis (UC), celiac disease, type 1 diabetes, and rheumatoid arthritis – all share a dysfunctional intestinal barrier early in disease, multiple studies addressed the impact of *PTPN2* alleles on epithelial barrier function. Yilmaz and others, for example, did this by monitoring the intestinal microbiome in patients and their data led to a model in which TCPTP-mediated dysfunction of autophagy, aberrant inflammasome activation and altered T-cell activation and differentiation lead to microbiota alterations and barrier defects (Yilmaz et al., 2018). Recently, Marchelletta and others (Marchelletta et al., 2021) provided a molecular explanation for defunct epithelial barrier function and tight junction organization as a consequence of dysfunctional TCPTP. They identified a role for TCPTP in the matriptase-mediated regulation of claudin-2 levels and tight junction stability, and suppression of cytokine-mediated JAK-STAT signals in the epithelial cells. CD patient samples homozygous or heterozygous for SNP rs1893217 displayed elevated claudin-2 and severely reduced TCPTP levels compared with samples carrying wildtype *PTPN2* alleles (Marchelletta et al., 2021). This is surprising since the rs1893217 SNP resides in intron 7 (c.858 + 4862T>C) of *PTPN2.* An earlier study had indicated that this *PTPN2* polymorphism did not affect ectopic baseline expression levels, but the finding that rs1893217 effects could be mimicked by siRNA-mediated silencing of *PTPN2* still led to the conclusion that the polymorphisms acts as a loss-of-function variation (Scharl et al., 2012). Thus, from the rs1893217 case we learn how difficult it is to predict phenotypic consequences of SNPs that are not in the gene’s protein coding regions.

### 2.3 *PTPN3* and *PTPN4*

The genes *PTPN3* and *PTPN4* encode enzymes that are characterized by an N-terminal FERM domain, a C-terminal PTP domain, and an intervening single PSD-95/Dlg/ZO-1 homology (PDZ) domain (Figure 1). The FERM domain enables submembranous interactions, as observed for founding members band 4.1, ezrin, radixin and moesin. PDZ domains represent one of the most prevalent ‘structural cassettes’ and they yield binding potential to C-terminal as well as internal protein sequences. Consequently, the proteins encoded by *PTPN3* and *PTPN4* (PTPH1 and PTP-MEG1, respectively) may be expected to regulate the functioning of submembranous protein scaffolds. PTPH1 is rather broadly expressed, whereas highest PTP-MEG1 levels are found in brain and in cells of the immune system. Although *in vitro* studies pointed to a regulatory role for these phosphatases in the immune response, notably in T-cell receptor signaling, this was not apparent in mouse knockout studies (Bauler et al., 2008; Patrignani et al., 2010). Additionally, PTP-MEG1 knockouts displayed behavioral abnormalities (Kina et al., 2007).

Several genomic variants have been recorded for *PTPN3* (Supplementary Table S1) but clear-cut phenotypic consequences remain to be revealed. The SNP rs3793524 in *PTPN3* was mentioned in a search for candidate genes in cleft lip/palate and dental anomalies as an additional affection status, but the linkage was marginal (*p*-value .04) and only observed in families that combined cleft lip and palate as well as dental anomalies (Vieira et al., 2008). The very same polymorphism also ended up in a panel of nonsynonymous SNPs that are believed to represent candidate genetic factors involved in breast cancer etiology and that may be critical for treatment outcome as well (Savas et al., 2006). Finally, a large exome sequencing study disclosed four schizophrenia patients that each carried a *de novo* missense mutation in a different PTP gene; either in *PTPN3* or in one of the three receptor-type PTP genes PTPRF, PTPRG and PTPRJ (Fromer et al., 2014). The identified SNP in *PTPN3* (c.1339C>A p.Q447K) is not yet covered by an rs-number, but HOPE-mediated analyses (Venselaar et al., 2010) suggests that the glutamine-lysine change at position 447 in PTPH1 may be without consequences because the mutant residue is present at this position in homologous sequences and thus seems tolerable. However, one cannot exclude that specific protein-protein interactions important for synaptic functionality may be jeopardized in the p.Q447K variant. Finally, WES has unveiled potential pathogenic variants in *PTPN3* (as well as in *PTPRC*, to be discussed later) involved in familial autoimmunity diseases (Wang et al., 2020).

**FIGURE 2:**
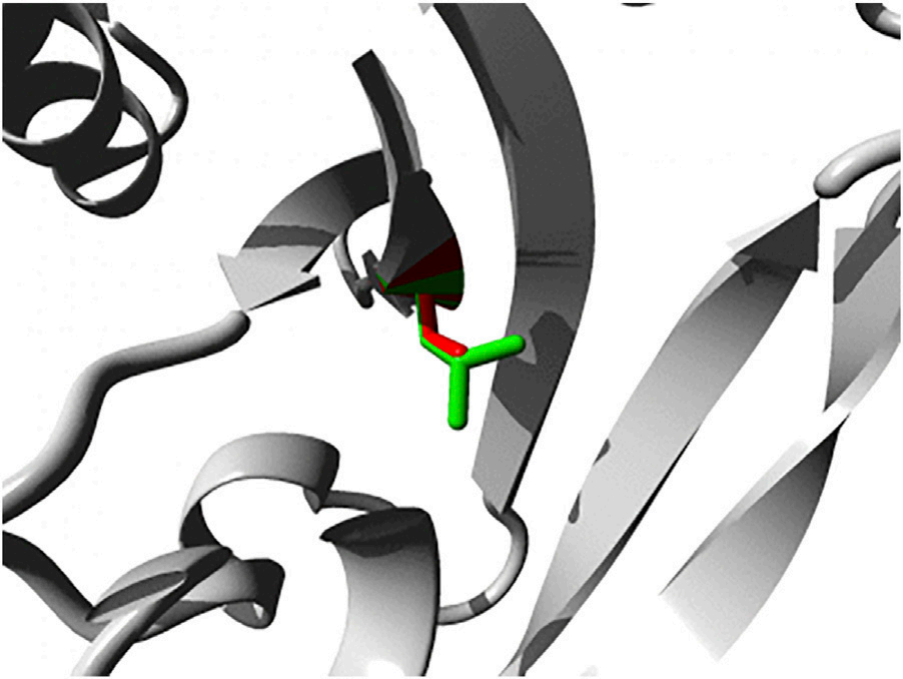
Close-up of the p.L72S mutation within the FERM domain of PTP-MEG1. Both the wild-type and mutant side chain are shown in green and red, respectively. The direct surrounding of the protein is shown in grey. Image was generated using HOPE (Venselaar et al., 2010).

Also *PTPN4* variants have been linked to neurodevelopmental pathologies (Williamson et al., 2015; Szczaluba et al., 2018). A *de novo* deletion of some 90–160 kbp, essentially removing the *PTPN4* gene only, was observed in the genomic DNAs of an identical twin with Rett syndrome-like features, but not in that of their parents or healthy siblings (Williamson et al., 2015). Rett syndrome is characterized by impairment or complete loss of language and hand skills, gait abnormalities, and stereotypic hand movements. Although many cases find their origin in mutations in the gene encoding methyl-CpG binding protein 2 (MECP2) several other disease genes that cause similar and overlapping syndromes have been identified as well. The list now also includes the one encoding PTP-MEG1 and expression data are in line with a role for MECP2 in *PTPN4* promoter regulation (Williamson et al., 2015). More recently, again a *de novo* mutation in *PTPN4* was detected in a patient with multiple developmental defects, autistic features and increased immunoglobulin E levels (Szczaluba et al., 2018). Interestingly, the PTP-MEG1 variant (c.215T>C p.L72S) displayed defective subcellular localization; ectopic expression of the pL72S mutant in cultured hippocampal neurons revealed its absence in dendritic spines as compared to wildtype PTP-MEG1. Substitution of the hydrophobic leucine at position 72, in the first of the three globular lobes that form the FERM domain, by a polar serine residue is likely affecting the intimate inter-domain contacts that enable FERM domains to bind proteins and/or lipids at the cell cortex (Pemberton and Balla, 2019). HOPE analyses indeed position the L72S structural change quite close to the shallow binding cleft located between the FERM subdomains (Figure 2). Recently six additional mutations in *PTPN4* were implicated to cause (neuro) developmental disorders (Chmielewska et al., 2021); five are documented in dbSNP (rs1677776998; rs1678083679; rs1678390512; rs1679218434; rs1259252500) and one (c.2171T>C p.I724T) is not, but all are predicted to disrupt PTP-MEG1 function (Chmielewska et al., 2021). Also in the ClinVar database *PTPN4* SNPs rs1679434397 (c.2491C>A, p.L831I) and, once more, rs1679218434 (c.1738G>T, p.D580Y) have been connected to intellectual disability and autism spectrum disorder (ASD), but functional evidence is currently lacking. Furthermore, also in a study of whole exome sequencing data an association for *PTPN4* sequence variants with developmental disorders, including brain and cardiac anomalies, small size, and dysmorphic features, was established (Lek et al., 2016). The gene also ranked amongst nonreceptor PTP genes that influence the risk of hepatocellular carcinoma development in hepatitis B-infected individuals (Shen et al., 2020a). Thus, although mouse *Ptpn4* knockout studies revealed subtle phenotypes, *PTPN4* represents a developmentally crucial gene in humans.

### 2.4 *PTPN6* and *PTPN11*

**FIGURE 3:**
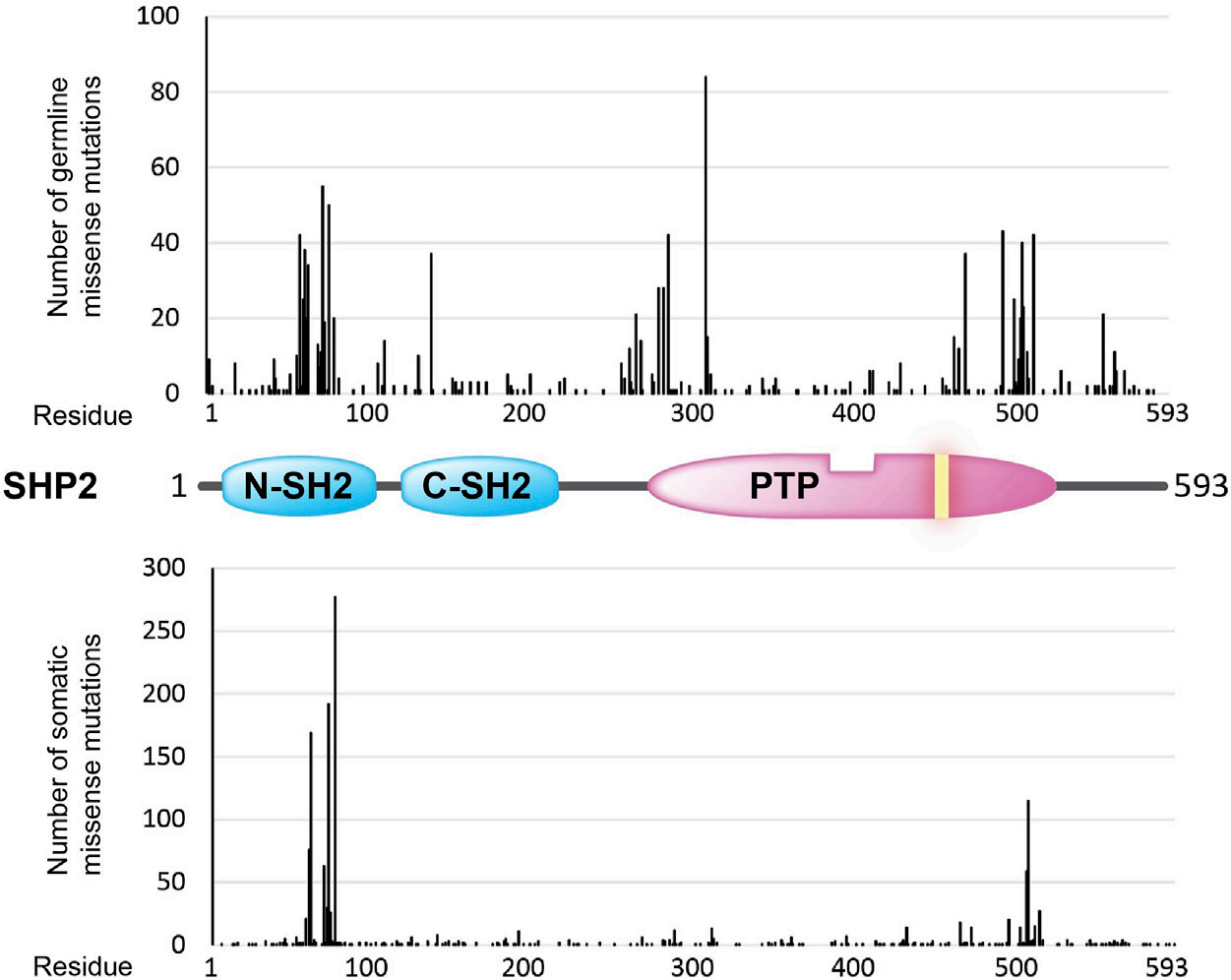
Overview of amino acid positions (*x*-axis) in the SHP2 protein (structural cartoon in the middle) that are found mutated (missense mutations) in the germline (top graph; ClinVar counts) and in sporadic cancer cases (lower graph; COSMIC counts). Occurrence frequencies (*y*-axis) are represented by the vertical black bars. The catalytic PTP domain and the Src homology type 2 (SH2) domains are depicted as in Figure 1. The yellow bar in the PTP segment indicates the position of the essential catalytic site cysteine.

*PTPN6* and *PTPN11* genes encode two highly-related PTPs (SHP1 and SHP2, respectively) containing a tandem of Src Homology 2 (SH2) domains, N-SH2 and C-SH2 (Figures 1, 3), a unique feature in the PTP family which confers to these two PTPs a high cell signaling regulatory potential. Upon binding of the N-SH2 domain in SHP1 and SHP2 to cognate phosphotyrosine-containing proteins, thereby terminating its intra-molecular auto-inhibitory effect, these PTPs become catalytically active and dephosphorylate specific protein substrates, which include a variety of receptors and adaptor signaling proteins (Dempke et al., 2018; Garg et al., 2020). Intriguingly, both PTPs can undergo liquid-liquid phase separation -a rather novel concept in molecular cell biology that opens myriad possibilities to dynamically compartmentalize intracellular reactions- and this ability, being sequence-dependent, may well contribute to the molecular etiology caused by the numerous SHP1/SHP2 sequence variants (Zhang et al., 2022).

SHP1 is predominantly expressed in hematopoietic cells, and due to an upstream alternative promoter also transcripts in epithelial cells are found (Banville et al., 1995). In mice *Ptpn6* mutations are responsible for the motheaten phenotype, a severe autoimmune and immunodeficiency syndrome (reviewed in (Kiratikanon et al., 2022)). In humans such an association is not documented in OMIM but extensive skin infiltration by neutrophils as found in Pyoderma gangrenosum (PG) and Sweets syndrome (SW), two uncommon autoinflammatory neutrophilic dermatoses, has been attributed in one study to deletions and a heterozygous p.E441G mutation in *PTPN6* (Nesterovitch et al., 2011a). More recently, a loss-of-function p.A455T heterozygous mutation has been reported in a family with early-onset emphysema (Bosse et al., 2019). Interestingly, the spontaneous insertion of a b2 type short interspersed repeat into *Ptpn6* exon six was found to result in decreased activity of the encoded enzyme and in an autoinflammatory mouse phenotype that resembles neutrophilic dermatoses in humans (Nesterovitch et al., 2011b). Detection of comparable genetic alterations in humans would reinforce the potential causative linkage between *PTPN6* defects and autoinflammatory diseases.

*PTPN11* codes for the homologous protein SHP2, which is ubiquitously expressed and a major positive regulator of the RAS/MAPK pathway. Consequently, SHP2 behaves as a pro-oncogenic protein in many cancer types, and *PTPN11* gain-of-function mutations are relatively frequent in human tumors, such as endometrium, hematopoietic/lymphoid, melanoma, and neuroblastoma tumors. The cancer-associated *PTPN11* mutations target two hotspots at the N-SH2 and PTP domains, respectively (Figure 3), that mostly result in an increased phosphatase activity or in a reduced threshold for SHP2 activation (Chan et al., 2008; Zhang et al., 2015). In contrast, SHP2 has been proposed to play a major tumor suppressive role in hepatocarcinogenesis (Li et al., 2012), in line with the finding that a short tandem repeat polymorphism of *PTPN11* (rs199618935, now merged into rs80269561) that leads to increased SHP2 levels confers a decreased risk to develop hepatocellular carcinoma (Zhao et al., 2014). Although alterations in SHP2 enzymatic activity seem to be directly related with human disease, Guo and Xu reviewed data indicating that phosphatase-independent, protein-protein interactive functions may also contribute (Guo and Xu, 2020).

*PTPN11* has been identified as a causative gene of Noonan Syndrome (NS), Noonan Syndrome with Multiple Lentigines (NSML, formerly known as Leopard Syndrome), Juvenile Myelomonocytic Leukemia (JMML), and Metachondromatosis (MC), all of which are clustered as different RASopathies due to sharing of alterations in the RAS/MAPK signaling pathway (Yang and Neel, 2013; Huang et al., 2014; Tajan et al., 2015; Liao and Mehta, 2019; Shen et al., 2020b; Dong et al., 2021). More than three hundred *PTPN11* variants are listed in ClinVar database. In NS and JMML, germline mutations at *PTPN11* replicate mostly the N-SH2 and PTP domain hot spots and SHP2 gain-of-function properties associated with sporadic tumors. *PTPN11* mutations associated with NSML, however, generate catalytically defective enzymes. It has also been proposed that some NSML mutations, such as p.Y279C and p.T468M, rather increase the affinity of SHP2’s SH2 domains for their phosphotyrosine-containing targets, thus making it more easy to activate the enzyme (Oishi et al., 2009; Yu et al., 2013). Interestingly, these two mutations have been found in hepatocellular carcinomas (COSMIC database), in which SHP2 has been proposed to act as a tumor suppressor (Li et al., 2012). *PTPN11* mutations linked to MC suggest haploinsufficiency, since the frameshift, nonsense, and splice-site mutations that create truncated SHP2 proteins occur heterozygotic (Bowen et al., 2011). Notably, these types of mutations are found with very low frequency in sporadic tumors (COSMIC database). The search for a genetic component in congenital heart disease cases revealed that also more subtle *PTPN11* variants may have pathological effects, i.e. on the spatio-temporal control of RAS-mediated signals during embryonic heart development (Xu et al., 2022).

The general distribution of *PTPN11* germline missense mutations differs from the distribution of *PTPN11* cancer-associated somatic mutations, with a mutation hotspot at the beginning of the PTP domain in the germline set of mutations that is not that clearly manifested in the set of somatic mutations (Figure 3). Whether this could be related with *PTPN11* mutation-specific pathogenic effects or with differences in embryonic lethality associated to the distinct sets of mutations is worth exploring. In this regard, strongly activating *PTPN11* mutations show decreased viability and embryonic lethality in mice (Araki et al., 2004).

In summary, a large array of different *PTPN11* gene alterations -from gene deletions to SNPs causing subtle changes in phosphotyrosine binding specificity and phosphatase activity-account for different human developmental and oncogenic disorders which have in common a defective regulation of the RAS/MAPK pathway. The involvement of SHP2 in cancer progression has boosted the identification and preclinical validation of SHP2 specific allosteric inhibitors which bind to closed inactive SHP2 blocking its conformational opening and activation, some of which are being tested in anti-cancer clinical trials (Yuan et al., 2020; Liu et al., 2021a). Given that JMML is a major cause of death in *PTPN11*-associated NS patients (Strullu et al., 2014) the possibility to treat *PTPN11* mutation associated RASopathies with SHP2 inhibitors also needs to be explored, although clinically suitable inhibitors which bind to active SHP2 would be required. Likewise, the elucidation of disease-specific SHP2 substrates or binding-partners that are involved in the pathogeny may further aid the design of therapies for SHP2-based RASopathies (Yi et al., 2022). In addition, this may have bearing for some Werner syndrome patients that, in addition to the homozygous deletion of the causative gene *RECQL2*, suffer from *de novo* activating mutations in *PTPN11* (Priolo et al., 2022).

### 2.5 *PTPN9*

**FIGURE 4:**
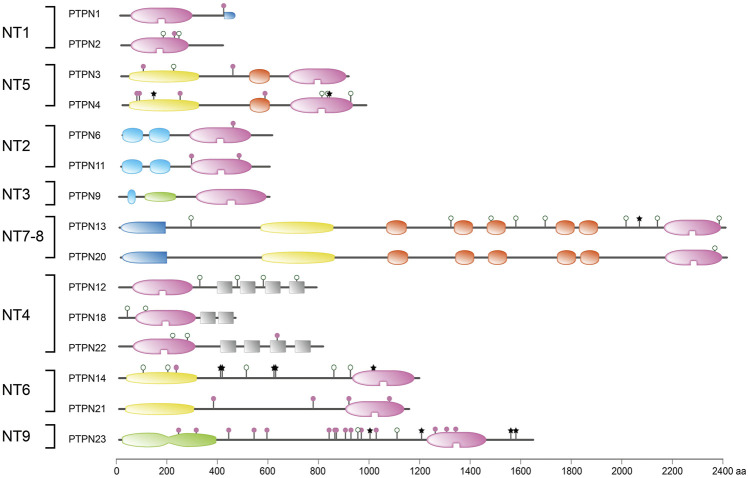
Protein sequence variant positions in non-transmembrane PTPs found in literature or arising from a common SNP. Classification (left) and protein domain structure build-up (right) of the ‘NT’ subclass of PTPs are as in Figure 1. PTPN5 and PTPN7 are not depicted here; they form part of the receptor-type subgroup R7. Sequence variants are represented by lollipops on the structural cartoons. The reddish purple lollipops represent non-synonymous mutants that are discussed in the text. The black star lollipops reflect frame-shift and non-sense variants discussed in the text. Lollipops with green open circles represent nonsynonymous variants occurring in >1% of the population and have not been linked to phenotypic consequences in the literature. All variant positions and linked corresponding literature references are included in Supplementary Table S1. The horizontal bar at the bottom indicates protein sizes.

The broadly expressed gene *PTPN9* encodes for a 593-amino acid protein, PTP-MEG2, that consists of a so-called Sec14 domain and C-terminally the catalytic PTP segment (Figure 1). The Sec14 module is homologous to protein domains that are known for their binding capacity towards metabolites like retinaldehydes and phosphatidyl-inositides, thus PTP-MEG2 activity could potentially be under the influence of hydrophobic ligands. Cellular studies point to a crucial contribution of PTP-MEG2 to vesicle fusion (Huynh et al., 2004) and exocytosis events (Xu et al., 2021), and the transport of transmembrane proteins, like the receptor tyrosine kinase TrkA, that participate in phosphotyrosine-dependent signals (Zhang et al., 2016a). *Ptpn9* knockout mice face serious developmental issues as almost all embryos die *in utero* and display a plethora of defects including vascular, bone and neural tube abnormalities (Wang et al., 2005). All this makes sense in the light of an important role in the secretory process, yet thus far hereditable human pathologies have not been assigned to *PTPN9* (as reflected in Figure 4). A decade ago the intronic SNP rs11635996 in *PTPN9* served in a search for candidate genes in four loci linked to a hereditable type of subclinical atherosclerosis that manifests through carotid plaque, but other genes on chromosome 15q exhibited stronger linkage (Dong et al., 2012). More recently, a large-scale meta-analyses of GWAS studies aimed at Alzheimer’s disease (AD) remained without genome-wide significant associations, but the combination with hippocampal transcriptomic data allowed for a transcriptome-wide association study that pointed to *PTPN9* as one out of 24 genes that could affect hippocampus-dependent AD development (Liu et al., 2021b). Three *PTPN9* SNPs were recently included in a search for genetic factors in Alcohol-induced osteonecrosis femoral head necrosis but no significant correlation was found (Xiong et al., 2022).

### 2.6 *PTPN12*, *PTPN18* and *PTPN22*

Proteins encoded by genes *PTPN12*, *PTPN18* and *PTPN22* (PTP-PEST, BPD1 and LYP, respectively) all contain the enzymatic PTP domain N-terminally and have C-terminal tails of varying lengths. These subtype NT4 PTPs are spiked with sequences rich in proline, glutamic acid, serine and threonine, known as PEST domains that enhance the protein’s turnover *via* proteasomal or calpain-mediated degradation (Rechsteiner and Rogers, 1996). Noteworthy, combined with sites for post-translational modifications (PTMs) such PEST regions are preferably found in intrinsically disordered protein regions (IDPRs). IDPR-containing proteins are considered crucial hubs in cellular processes that need tight regulation through PTM and protein turnover, and aberrant regulation of IDPs/IDPRs thus contributes to various human diseases (Darling and Uversky, 2018), as exemplified by the C-terminal region of the tumor suppressor PTEN (Pulido, 2015). Indeed, at least for one of the PEST-containing PTPs, LYP, this connection has been firmly established.

PTP-PEST is a broadly expressed protein of 780 amino acid residues that has four of those PEST domains. It is a developmentally essential gene since *Ptpn12* knockout mice die *in utero* around day 10, and multiple studies using cell models and conditional mouse mutants underscored important functions in adult tissues as well (reviewed in (Lee and Rhee, 2019)). Also the frequency of *PTPN12* mutations in tumor samples illustrates the protein’s impact. Irrespective, a monogenic disease connotation for PTPN12 is yet to be disclosed. Candidacy as a contributor to heritable colorectal cancer susceptibility (de Voer et al., 2016; Belhadj et al., 2020) and recently the risk to develop hepatocellular carcinomas has been reported (Shen et al., 2020a), but a meta-analysis of proposed colorectal cancer predisposition genes rather called for caution in claiming cancer risk candidacy, including that of *PTPN12* (Broderick et al., 2017; Terradas et al., 2020). There is a single mentioning of *PTPN12* involvement in enthesitis-related arthritis (Weiss, 2016) but we suspect that *PTPN22* was meant here. Finally, in a genome-wide association (GWA) meta-analysis of the genetic contribution to personality trait variation in Koreans the *PTPN12* SNP rs12537271 was found most highly associated with extraversion, one of the five dimensions of personality (Kim et al., 2015). However, the genetic association (*p* = 1.47 × 10^–7^) did not reach the genome-wide significance threshold (*p* < 5 × 10^–8^).

*PTPN18* encodes the shortest of the PEST-containing PTPs; BPD1 is 459 residues long and harbors a single PEST region. Its expression is mostly in brain and in epithelial cells, and shortly after its discovery BPD1 was recognized for its capacity to interact with receptor tyrosine kinase ERBB2, thereby not only determining the receptor’s phosphorylation status but also its intracellular fate following endocytosis (Wang et al., 2014a). *PTPN18* was amongst the three genes that were identified in a genetic study that compared mouse quantitative trait loci determining insulin secretory performance of isolated pancreatic beta cells with human diabetes-related SNPs (Keller et al., 2019). Indeed thousands of SNPs are noted for the gene but the 41 variants that are included in ClinVar in fact involve deletions or duplications of multiple genes in the area, leaving *PTPN18*’s contribution to be revealed. Recently, using an impressive bioinformatics pipeline, Yan and co-workers identified *PTPN18* as one of the 26 candidate genes for the common congenital birth defect cleft lip with or without cleft palate (Yan et al., 2020). Further tests using mouse models will be required to substantiate this finding.

The expression of LYP (a.k.a. PTPN22) the largest of the three PEST-containing PTPs (807 residues long, carrying some five PEST domains), is limited to lymphoid cells. This is perfectly in line with the numerous *PTPN22* risk alleles for effectively all autoimmune diseases: type I diabetes mellitus, rheumatoid arthritis, Hashimoto thyroiditis, Graves disease, systemic lupus erythematosus (SLE), familial hypoadrenocorticism, Addison’s disease, psoriasis, Anti-neutrophil cytoplasmic autoantibody (ANCA)-associated vasculitis, and primary immune thrombocytopenia (Armitage et al., 2021; Tizaoui et al., 2021). Also studies on *Ptpn22* knockout mice underscore its involvement in innate and adaptive immunity and its negative regulatory role in T-cell receptor (TCR) signaling (Hasegawa et al., 2004). Some recent reviews present comprehensive recollections of the genetic data underscoring LYP’s involvement in immune disorders (Armitage et al., 2021; Tizaoui et al., 2021; Royrvik and Husebye, 2022) and due to space restrictions we refer the reader to those papers. Also a meta-analysis of the association of gene *PTPN22* (notably SNPs rs2476601 and rs2488457) with susceptibility of primary immune thrombocytopenia appeared very recently (Tian et al., 2022). Intriguingly, *PTPN22* variants may -also- affect the function of an overlapping gene that produces a transcript known as AP4B1-AS1. We limit ourselves therefore to recapitulating the conclusion compiled from all underlying studies; that the mechanisms how disease-associated *PTPN22* variants affect innate and acquired immunity is heavily dependent on the cell types studied. Furthermore, conflicting findings regarding the effect of the major *PTPN22* disease allele (p.R620W) between mouse and human studies currently prohibit clear answers to such questions. Because the *PTPN22* orthologs between these species are too divergent (Armitage et al., 2021), future studies will rely on primate models.

### 2.7 *PTPN13* and *PTPN20*

The proteins PTPN13 and PTPN20 were originally viewed as members of two separate non-transmembrane PTP classes (NT7 and NT8, respectively) but, triggered by studies in zebrafish, den Hertog and coworkers noted that gene *FRMPD2* in fact represented ‘the missing upper half’ of the *PTPN20* gene (van Eekelen et al., 2012). The elongated human *PTPN20* gene then gives rise to a transcript that encodes a full paralog of PTPN13; both PTPs contain a KIND, a FERM, and five PDZ domains in addition to the C-terminal catalytic domain (Figure 1). In addition, some 500 kbp downstream of PTPN20 another, probably pseudogene, copy of the PTPN20 gene has been mapped (Fodero-Tavoletti et al., 2005). *PTPN13* and *PTPN20* transcripts undergo extensive alternative splicing, generating multiple isoforms with different protein interaction domain compositions. The resulting modular use of anchoring and scaffolding possibilities can be held responsible for the many protein interactions and functional connotations that these largest PTPs received over the years.

Gene *PTPN13* spans 48 exons that encode for an almost 2,500 amino acids long enzyme that, due to its FERM domain (Figure 1), localizes in submembranous areas in many cell types. In polarized epithelia highest levels are found at the apical membrane side, but PTPN13 localization is dynamic and the protein even shuttles in and out of the nucleus (McHeik et al., 2020). Oddly enough, its potential to interact with a plethora of signaling and structural proteins and the observed gene mutations and translocations in cancer specimens for the gene have resulted in a ‘split personality’; depending on the context an oncogenic but also a tumor suppressive attribution to PTPN13 can be made (McHeik et al., 2020). Knock-out mouse studies have not enlightened this picture, but for the many SNPs some correlations to human pathologies have been postulated. In ClinVar 23 SNPs are directly linked to *PTPN13* as the sole gene, and 19 represent missense mutations. For rs10033029 (p.F1356L), rs2230600 (p.I1522M) and rs989902 (p.Y2081D) an association with the risk to develop epithelial cancers was established for multiple cohorts (Yeh et al., 2006; Mita et al., 2010; Wei et al., 2013) but for the majority their impact has not been revealed yet.

Hirschsprung disease (HSCR) is a disorder of the enteric nervous system with an incidence of one in 5K live births, and mutations in over a dozen different genes still account for only a subset of cases. Some years back, in addition to a probably disease-causing *de novo* mutation in the proto-oncogene *RET*, also a protein-truncating *PTPN13* variant (p.W2132*) was detected in a single HSCR patient’s DNA (Zhang et al., 2017). Given PTPN13’s pronounced expression in normal colon and gut tissue, authors probed for a contributing role of the phosphatase in HSCR etiology, but no further *PTPN13* mutations were apparent in the cohort of 83 patients, and PTPN13 protein levels appeared normal in colon tissue from 16 of them (Zhang et al., 2017). In that same year, a study reported on the PDZ-domain-mediated interaction of PTPN13 with calpain-2 but not with calpain-1 (Wang et al., 2017). These two calpains are the major calcium-dependent cysteine proteases in brain and play opposing roles in synaptic plasticity and neuronal survival. Intriguingly, the authors could show that PTPN13 serves as a substrate for calpain-2, which cleaves the giant phosphatase at sites just before PDZ3 and/or slightly downstream of PDZ5, effectively releasing the PTP moiety from the anchoring domains of the protein. They did not detect stable C-terminal PTPN13 breakdown products containing the PTP domain after cleavage, implying that the calpain-2-mediated cleavage in fact inactivates the phosphatase. Given that tau hyperphosphorylation is a hallmark of several neurological disorders, including Alzheimer’s disease (AD) and traumatic brain injury (TBI), the authors tested whether they could link TBI-induced calpain-2 activation, *via* PTPN13 cleavage, to tau hyperphosphorylation. Indeed, using cell and mouse models, they provided evidence for a calpain-2-PTPN13-phospho-tau pathway that could have bearing for tangle formation, AD development and related neurological disorders (Wang et al., 2017).

Intriguingly, both the p.W2132* truncating variant and the calpain-2 mediated cleavage of PTPN13 both produce protein halves that are reminiscent of the products to be expected from the separated *FRMPD2* and *PTPN20* genes as they were viewed originally (van Eekelen et al., 2012). Unfortunately, the information on PTPN20 and its putative pseudogene (Fodero-Tavoletti et al., 2005) is rather minimal in literature and databases. We found one report, on the two most common neurodegenerative disorders (Alzheimer’s disease and Parkinson’s disease), that used single cell RNA-seq data and computational pipelines in order to identify novel genes and pathways whose activity is intrinsically altered in diseased brain (Bordone and Barbosa-Morais, 2020). PTPN20 turned out to be amongst the genes whose expression was differentially altered with cellular composition in AD brains compared with non-AD samples. However, no reports have linked *PTPN20* to neurological disorders thus far, and also for the nonsynonymous SNP rs202027139 (p.R392H) such a connotation is currently not apparent.

### 2.8 *PTPN14* and *PTPN21*

Like PTPN13 and PTPN20, the proteins PEZ and PTPD1 –encoded by genes *PTPN14* and *PTPN21*, respectively–also possess a FERM domain. However, KIND and PDZ domains are absent in these two PTPs (Figure 1) and instead acidic regions and putative SH3 domain-binding, proline-rich sequences can be discerned. PEZ expression is quite broad but absent in brain and liver. PTPD1 is expressed in multiple tissues with highest levels in lung, skeletal muscle, and placenta. Intriguingly, the catalytic domains of PEZ and PTPD1 did not display phosphatase activity against a broad panel of phosphopeptides (Barr et al., 2009), yet phosphotyrosine-containing substrate proteins have been identified for these enzymes (Wadham et al., 2003; Cardone et al., 2004). This may point to a rather context-dependent substrate selectivity of the PTPs, with the lipid-interacting FERM domain at their N-terminus perhaps as an important specificity determinant.

In the PEZ middle portion two PPxY motifs are present that facilitate binding to WW domain containing proteins. As a result, several components of the Hippo signaling pathway were found to interact with PEZ, posing the PTP as negative regulator of YAP/TAZ signaling (Sarmasti Emami et al., 2020). Furthermore, gene *PTPN14* turned out to be one of the targets for transcription factor p53. Analyses of pancreatic cancer material revealed that *TP53* and *PTP14* mutations are mutually exclusive and that both genetic changes lead to enhanced YAP signals, suggesting a p53/PEZ/YAP pipeline that is crucial for tumor suppression (Mello et al., 2017). Other targets for PEZ are functioning at intercellular junctions and impact on angiogenesis and epithelial-mesenchymal transitions during development and in cancer (Wadham et al., 2003; Fu et al., 2020). Furthermore, PEZ is able to inhibit the process of metastasis by regulating secretory vesicular transport in cancer cells (Belle et al., 2015). It should therefore not come as a surprise that *PTPN14* variants have been linked to cancer predispositions (Zhang et al., 2016b; Olafsdottir et al., 2021) as well as malformations, including gingival fibromatosis with distinctive facies (Cogulu et al., 2021), lymphedema-posterior choanal atresia syndrome (Au et al., 2010; Bordbar et al., 2017) and hereditary hemorrhagic telangiectasia 1 (Benzinou et al., 2012; Letteboer et al., 2015). Quite recently, *PTPN14* nonsense mutations in the benign cutaneous neoplasm trichilemmoma (Russell-Goldman et al., 2022) were added to this list.

The structural similarities between PEZ and PTPD1, the two non-transmembrane type 6 PTPs, are also evident from the fact that the catalytic domain of both is targeted by the CR3 domain in the E7 protein of human papillomavirus 18, a protein segment that destines its interactors for proteasomal degradation (Lee et al., 2021). Surprisingly, whereas PEZ is known for its tumor suppressive function -in line with having an oncogenic HPV variant working towards inhibition of PEZ activity- PTPD1 is not degraded after complexing with the E7 protein. Furthermore, PTPD1 contributed significantly to migratory and invasive behavior of cancer cells, thus corroborating its candidacy as tumor promoting protein (Lee et al., 2021). As yet it is unclear what causes the two PTPs taking such opposite sides in tumor etiology, urging for more studies addressing their intracellular contact points. Intriguing roles for PTPD1 have been uncovered in neural developmental processes, especially survival signaling and, *via* its FERM domain, intracellular vesicle trafficking (Plani-Lam et al., 2015; Siddiqui et al., 2019). These findings provide mechanistic support for the associations that have been established for PTPN21 gene variants as modulators in Alzheimer’s disease (Park et al., 2017; Zhao et al., 2019) and schizophrenia (Chen et al., 2011).

### 2.9 *PTPN23*

The gene *PTPN23* encodes a large and rather unique enzyme characterized by the presence of a BRO1-like domain (Figure 1), a segment that is homologous to the yeast vacuolar sorting protein Bro1 and a human regulator of endosomal sorting named ALIX. In addition, in between the N-terminal BRO1 and C-terminal PTP domains, multiple putative SH3 domain binding motifs within a so-called His domain can be discerned. Expression is readily detected in epithelial cells of adult tissues, and the gene is also transcribed early during embryogenesis; knock-out of the mouse ortholog is lethal (Gingras et al., 2009). The catalytic phosphatase activity of the protein encoded by *PTPN23*, HD-PTP, has been subject of discussion over the years. The presence of S instead of A in the catalytic site motif VHCSAGXGRTG, and E instead of D in the conserved WPD loop, both characteristic sequence elements for the PTP superfamily, suggested it may be a pseudophosphatase. HD-PTP’s crucial role in ephrin-mediated signaling indeed was unaffected by substituting the catalytic site cysteine that is essential for phosphatase activity by classical PTPs (Lahaie et al., 2019). Nevertheless, indications of low enzyme activity and/or extremely selective substrate specificity have been gathered (Lin et al., 2011) and, irrespective of PTP activity, HD-PTP has been shown to be involved in cell adhesion and migration processes, notably endosomal trafficking, ciliogenesis, and assembly of spliceosome components (Lin et al., 2011; Ali et al., 2013; Smigiel et al., 2018; Lahaie et al., 2019; Bend et al., 2020).

Over three hundred SNPs have been reported for *PTPN23* in the ClinVar database. Regarding the phenotypic consequences, compelling cases for *PTPN23* association with neurodevelopmental disorders and epilepsy have been documented. For example, in NEDBASS (neurodevelopmental disorder and structural brain anomalies with seizures and spasticity) patient materials several different nonsense and missense mutations in HD-PTP have been detected (Alazami et al., 2015; Sowada et al., 2017; Trujillano et al., 2017; Smigiel et al., 2018; Bend et al., 2020). Also in one pediatric epilepsy patient, using whole exome sequencing, a *PTPN23* mutation was found (Rochtus et al., 2020) supporting its candidacy as epilepsy-associated gene (Alazami et al., 2015). One should note, however, that most of the detected gene variants have not been subjected to functional studies. A recent report on hereditary spastic paraplegia (HSPs), for example, revealed that some of these *PTPN23* alterations are likely benign and that biallelic alterations in the gene underly the heterogeneity of the complex HSP clinical spectrum (Khalaf-Nazzal et al., 2021).

### 2.10 *PTPN5, PTPN7* and *PTPRR*

**FIGURE 5:**
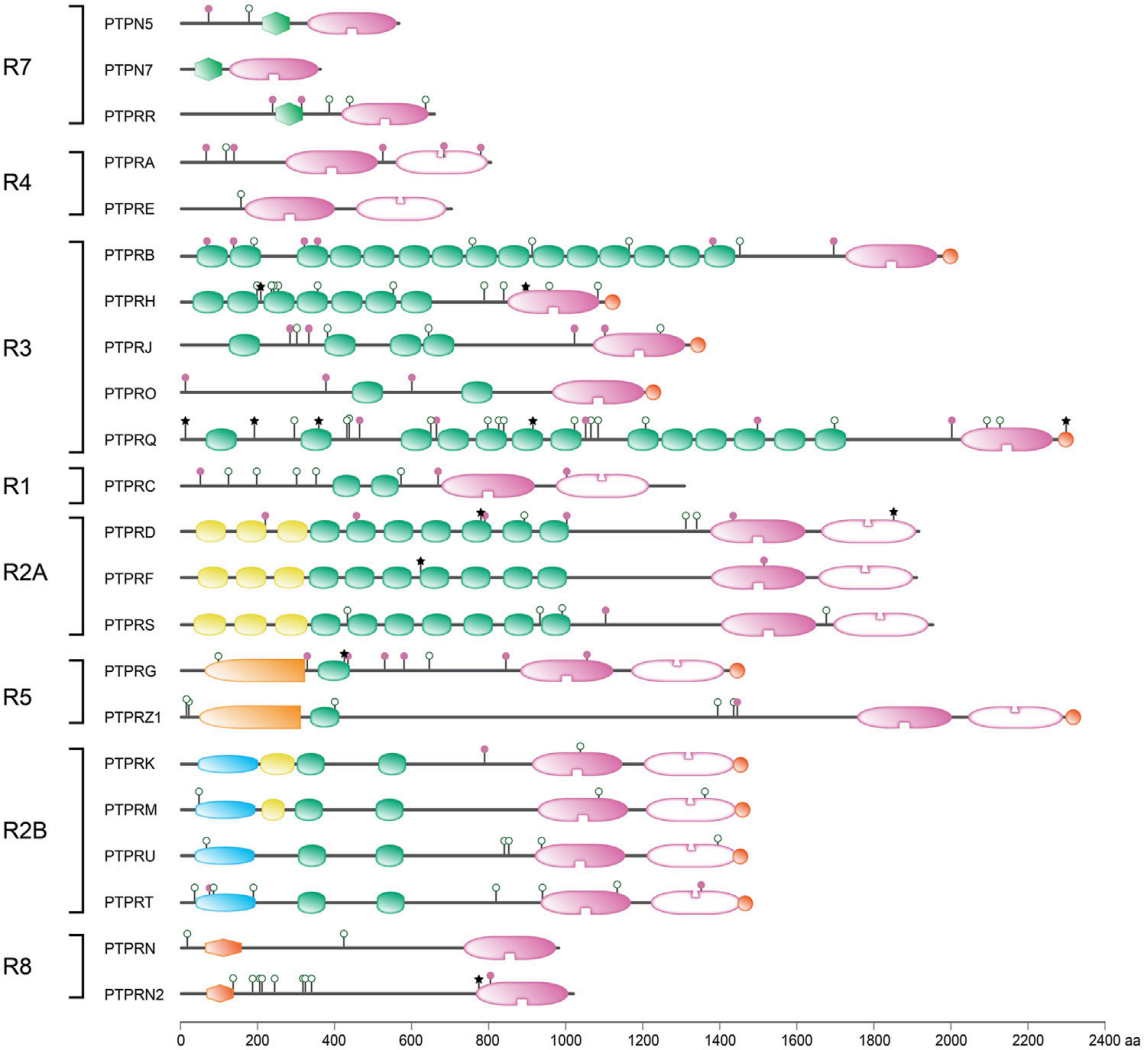
Protein sequence variant positions in receptor-type PTPs found in literature or arising from a common SNP. Classification (left) and protein domain structure build-up (right) of the ‘R’ subclass of PTPs are as in Figure 1. PTPN5 and PTPN7 are included here as members of receptor-type subgroup R7. Sequence variants are represented by lollipops on the structural cartoons. The reddish purple lollipops represent non-synonymous mutants that are discussed in the text. The black star lollipops reflect frame-shift and non-sense variants discussed in the text. Lollipops with green open circles represent nonsynonymous variants occurring in >1% of the population and have not been linked to phenotypic consequences in the literature. All variant positions and linked corresponding literature references are included in Supplementary Table S1. The horizontal bar at the bottom indicates protein sizes.

At the time that the first PTP sequences were uncovered it made sense to divide the family in receptor-type, transmembrane PTPs and non-transmembrane members. In retrospect this now is confusing since there are multiple PTP genes that encode both receptor-type and non-transmembrane isoforms, either using alternative transcription start sites, alternative splicing and/or proteolytic processing. The so-called R7 subgroup of classical PTP genes, with members *PTPN5, PTPN7* and *PTPRR*, entails such a merger of intracellular and membrane-spanning PTP isoforms (Alonso and Pulido, 2016). The reason to group these three genes is the shared unique sequence feature found in the encoded protein isoforms; just N-terminal of the catalytic PTP domain they carry a so-called kinase-interacting motif (KIM) (Barr and Knapp, 2006). This KIM domain enables these PTPs to interact specifically with serine/threonine kinases of the MAPK family (Pulido et al., 1998), an association that can be blocked through phosphorylation of a serine residue within the KIM by cyclic-AMP-dependent protein kinase (PKA) (Blanco-Aparicio et al., 1999). The KIM domain is not exclusively present in the R7-type classical PTPs; also many dual-specificity PTPs exploit the module and are known as MAP kinase phosphatases (Dickinson and Keyse, 2006). In addition to the KIM domain the R7-type PTPs also contain a sequence stretch named KISS, for kinase-specificity sequence, that is instrumental for the MAPK preference displayed by the members (Munoz et al., 2003). Since MAPK proteins act downstream of growth factors and their receptors the KIM-containing PTPs are predicted to impinge on all cell growth, differentiation and survival signaling pathways thinkable. One should not get the impression, though, that individual KIM-PTP genes are dispensable and that redundancy ensures fail-safe operation of these signaling circuitries. For example, in a study towards the cause of corticosteroid insensitivity in severe asthmatics, the knock-down of *PTPRR* resulted in elevated Ser226 phosphorylation and reduced nuclear translocation of glucocorticoid receptors, whereas silencing of *PTPN5* or *PTPN7* had no effect (Kobayashi et al., 2016). The examples below indeed underscore proven and potential disease links for *PTPN5*, *PTPN7* and *PTPRR* gene variants. An overview of variants that impinge on the respective proteins - thus the non-synonymous, nonsense and frame-shift mutations—is given in Figure 5.

Gene *PTPN5* is mainly expressed in the central nervous system and has the potential to result in the production of two major and two minor protein isoforms that are named STEP, for striatal-enriched PTP, followed by a number that reflects their apparent molecular weight (Kamceva et al., 2016). STEP_61_ and STEP_46_ represent the two major isoforms and the additional N-terminal segment in STEP_61_ contains hydrophobic stretches that anchor this isoform to the membrane. The minor isoforms, STEP_38_ and STEP_20_, lack the catalytic PTP domain but still contain the KIM and thus may modulate substrate accessibility for the major isoforms. STEP knockout mice have no apparent morphological defects, but detailed studies revealed a collection of subtle cognitive and behavioral abnormalities ((Olausson et al., 2012) and references therein). Additional studies corroborated that, besides MAPKs, also the NMDA and AMPA glutamate receptors represent prime STEP substrates (Carty et al., 2012). Dephosphorylation of subunits of these postsynaptic receptors triggers their endocytosis, hence determines synaptic functioning. Reports on the association of *PTPN5* gene variants with schizophrenia and cognition (Pelov et al., 2012) and Alzheimer’s disease (Cheng et al., 2018) adds to the evidence connecting STEP with neurologic and neuropsychiatric disorders (Mahaman et al., 2021). Most indications, thus far, come from compelling studies in various animal model systems, and using human post-mortem materials or iPSCs, that reflect schizophrenia (Carty et al., 2012; Pelov et al., 2012; Xu et al., 2018), Parkinson’s disease (Kurup et al., 2015), Fragile X syndrome (Chatterjee et al., 2018), Huntington’s disease (Garcia-Forn et al., 2018) and stress-related psychiatric disorders (Yang et al., 2012). Finally – and quite surprisingly, given its neuronal expression pattern - the rare missense variant rs56234898 in *PTPN5* was found to be significantly associated with a decreased severity of hypertrophic scarring following deep burns (Sood et al., 2016). To link STEP to cutaneous wound healing seems a long shot, but since the phosphatase is able to inhibit the stress MAPK p38, and experimental inhibition of p38 results in decreased fibrogenesis, one should definitively investigate potential *PTPN5* expression in peripheral nervous tissue as well.

*PTPN7* is regarded as rather tissue-specific; it is expressed in cells of the hematopoietic system. The encoded protein, HePTP, negatively regulates T-cell receptor signals by targeting the downstream MAPKs ERK and p38 (Oh-hora et al., 1999; Saxena et al., 1999). Knockout mouse studies, however, revealed that HePTP deficiency only resulted in subtle alterations in the spatiotemporal pattern of MAPK signals in hematopoietic cells when stimulated *ex vivo* (Gronda et al., 2001). Given the broad collection of MAPK phosphatases amongst the dual specificity PTPs (Dickinson and Keyse, 2006) this may be explained by ample compensation through other PTP superfamily members. The same reason may underlie the fact that an original observation in Vietnamese-Korean families, finding the intron variant rs10920338 in *PTPN7* being significantly associated with early childhood body length (Kim et al., 2010), has not been followed up in the literature.

As for *PTPN5*, also the gene *PTPRR* encodes many different protein isoforms (https://atlasgeneticsoncology.org/gene/41937/ptprr-(protein-tyrosine-phosphatase-receptor-type-r)). The longest one is a canonical single-pass transmembrane PTP that, after removal of the N-terminal signal peptide from the precursor protein, appears as a 71 kDa species. It explains why the gene was grouped among the ‘receptor types’ within the classical PTP subfamily (Figure 1). This receptor-type PTPRR isoform can additionally be post-translationally cleaved by furin-like proteases, rendering a 59 kDa PTP isoform. This cleavage site is also present in the 60 kDa isoform that results from alternative promoter use; this protein lacks an obvious signal peptide preceding the transmembrane segment, reminiscent of STEP_61_, and apparently behaves as a type III transmembrane molecule Additional PTPRR isoforms mimic the structure of the STEP_46_ isoform (Hendriks et al., 2009). *PTPRR* expression is rather tissue specific and highest in neuronal and neuroendocrine cells. Studies in mice that lack PTPRR phosphatase activity clearly demonstrated the impact of the KIM domain in all isoforms; increased phospho-MAPK levels were observed in brain extracts and animals displayed significant defects in their fine motor coordination and balance skills although morphological defects were absent (Chirivi et al., 2007). Behavioral studies highlighted subtle alterations in cerebral processes, notably in object recognition and exploratory tasks (Erkens et al., 2014), and studies focusing on the cerebellum revealed that PTPRR deficiency obstructed the MAPK-dependent positive feedback loop required for long-term depression at Purkinje cell synapses (Erkens et al., 2015). It is therefore difficult to fathom how the human *PTPRR* variant rs73341069 could become associated (currently, exome-wide significance is lacking) with the risk to develop prostate cancer (Rand et al., 2016), also because the consequent valine to isoleucine change within the transmembrane domain seems well tolerable. Actually, it seems more logical to expect an association for *PTPRR* with neurological pathologies. A decade ago *PTPRR* variant rs1513105 was indeed linked to increased occurrence of major depressive disorder in females of the Chinese Han population (Shi et al., 2012), a finding that may be substantiated by *PTPRR* overexpression studies in mice that revealed depressive-like symptoms as a response to chronic mild stress (Li et al., 2016) if one assumes that the intronic variant results in increased expression levels for the gene.

Dopamine signaling defects in the retina can lead to myopia, a rather common vision-threatening disorder, and since MAPK signaling is downstream of dopamine receptors a disease association for MAPK phosphatases may be expected. Genetic studies on high-grade myopia indeed pointed to a strong association with a missense mutation (rs3803036) in *PTPRR* (Hawthorne et al., 2013) and meta-analyses of *PTPRR* variant rs11178469 also revealed a relationship with visual refractive errors (Tedja et al., 2018). However, these associations of myopia or ocular parameters with *PTPRR* were not supported by studies involving Japanese (Yoshikawa et al., 2014) and Chinese (Xiao et al., 2021) cohorts. Expansion of data and inclusion of additional ethnic groups may clarify this putative contribution of *PTPRR* to myopia risk factors.

### 2.11 *PTPRA* and *PTPRE*

Also for the receptor-type PTP genes *PTPRA* and *PTPRE* their classification as ‘type IV transmembrane PTPs’ needs to be viewed more flexible. Although for *PTPRA* the reported isoforms are all single-pass transmembrane proteins that merely differ in their glycosylation and proteolytic processing status, gene *PTPRE* is able to yield both receptor-type and intracellular isoforms through the use of distinct promoters, alternative translation start codon use, and proteolytic events ((Gil-Henn et al., 2001) and references therein). Unlike the *PTPRR*-encoded proteins, however, *PTPRA* and *PTPRE* protein products do not contain a KIM domain. Rather, in contrast to the single PTP domain in the PTPRR transmembrane isoform, they carry a tandem set of PTP domains at their C-terminus, as is the case for most receptor-type PTPs (Figure 1). Intriguingly, these second, membrane-distal PTP domains usually display no – or only limited – enzymatic activity and are thought to modulate the substrate specificity and/or phosphatase activity of the first. Notably, structural studies on PTPRA have been fundamental in establishing a PTP regulatory concept based on a so-called wedge domain that is present in between the transmembrane segment and the proximal PTP unit and that would be responsible for dimer-induced receptor-type PTP inactivation (Jiang et al., 1999). Alternative ways to regulate phosphatase activity *via* inter-molecular or intramolecular interactions of both PTP domains have also been encountered, both for enzymes with and without wedge domain-like sequences (Hendriks et al., 2018).

Despite the obvious similarity in sequence and structure, PTPRA and PTPRE enzymes have distinct functions as was revealed by comparing phenotypes of knockout mouse models, for example on bone formation and osteoclast functioning (Finkelshtein et al., 2014). PTPRA is ubiquitously expressed and its interactions with key signaling molecules, including a plethora of tyrosine kinases and adaptor proteins, have linked it to adhesion, cell motility and cytoskeletal dynamics. Based on their observations in PTPRA-deficient mice and the notion that the human gene resides in a locus that is linked to psychotic illness, Sap and coworkers looked for an association of *PTPRA* variants with neuropathologies (Takahashi et al., 2011). Indeed, for SNP rs1016753 they detected a link with schizophrenia, and reduced *PTPRA* mRNA levels were noted in *postmortem* brain specimens of schizophrenia subjects. A subsequent study, involving resequencing and association analyses of the *PTPRA* locus in different cohorts of schizophrenia and autism spectrum disorder patients, did yield rare gene variants that may impair protein function but a significant genetic association with these pathologies could not be established (Xing et al., 2014). Recent whole exome sequence data, however, added new support, in the form of six new potentially damaging missense mutations, for a connection between *PTPRA* variants and schizophrenia (John et al., 2019). Additional genetic data should depict a more refined picture on *PTPRA’s* disease risk contribution. Furthermore, there is quite some molecular work to do in converting these new ‘potentially damaging’ mutations into functionally annotated SNPs. Moreover, one should keep an open eye for a connection of *PTPRA* variants with other pathologies as well. As an example we mention its recently suggested ties with HIV-1C acquisition and pathogenesis (Shevchenko et al., 2021), although the small size of the studied cohort implies the need for follow-up studies on *PTPRA* as a determinant in viral transmitted diseases.

In contrast to *PTPRA*, *PTPRE* is much more tissue-specifically expressed, with highest levels in brain and testis for the receptor-type isoform, and in thymus, spleen and hematopoietic cells for the cytoplasmic variant (reviewed in (Liang et al., 2019)). Regarding substrate specificity the *PTPRE*-encoded enzymes share many of the interacting proteins and substrates with *PTPRA*, including the cytosolic Src-family of tyrosine kinases. A recent transcriptome-wide association study pointed to *PTPRE* as one of the genes associated with autism spectrum disorders (Rodriguez-Fontenla and Carracedo, 2021). Another indication that appropriate *PTPRE* expression levels may be critical for well-being comes from a study aimed at detecting copy number variants that underly congenital heart disease (Glessner et al., 2014). Interestingly, *PTPRE* transcription control is in part exerted by a long non-coding transcript (lncRNA) that maps to the reverse strand of the gene itself (Han et al., 2019). This lncRNA PTPRE-AS1 enhances *PTPRE* gene expression in M2-type macrophages, *via* the binding of transcription factors that epigenetically control histone H3 trimethylation at the *PTPRE* promoter region, and as such represses IL-4-induced macrophage activation. Because CRISPR-Cas9-mediated inactivation of PTPRE-AS1 partially protected mice from induced acute colitis but also exacerbated induced pulmonary allergic inflammations (Han et al., 2019), this suggests a lncRNA-regulated role for *PTPRE* in the pathogenesis of inflammatory disease. To place this in perspective, there is also the report that a rather complex three-way chromosomal rearrangement disrupting *PTPRE* and three other genes remained without pathological consequences (Aristidou et al., 2018).

### 2.12 *PTPRB, PTPRH, PTPRJ, PTPRO* and *PTPRQ*

A clearly separated group of receptor-type PTPs is encoded by five genes that all result in transmembrane proteins with six to seventeen fibronectin type III (FNIII) repeats in their extracellular segment and a single phosphotyrosine phosphatase domain intracellulary; *PTPRB*, *PTPRH*, *PTPRJ*, *PTPRO* and *PTPRQ* (Figure 1). Evolutionary analysis predicts that *PTPRQ* branched off early during metazoan diversification from the common precursor of *PTPRB*, *PTPRH*, *PTPRJ* and *PTPRO*, in line with the finding that the former encodes an RPTP that dephosphorylates inositol phosphate rather than phosphotyrosine amino acids (Chicote et al., 2017a). The other four encode proteins (termed RPTPβ, SAP-1, CD148 and GLEPP1, respectively) do take phosphoproteins as substrates, and using artificial systems they all were able to bind and dephosphorylate the insulin receptor. In real life, however, their tissue-specific expression contributes to substrate selectivity. RPTPβ is most prominent in endothelia, SAP-1 is mainly in the intestine, and GLEPP1 expression is highest in brain and kidney. In contrast, CD148 is broadly expressed and likely represents the physiological R3-type phosphatase to counteract insulin signals (Shintani et al., 2015).

The endothelial expressed gene *PTPRB* encodes RPTPβ that has the vascular growth factor receptors as prime targets and thus is important for angiogenesis. This is not only substantiated by data studies involving mutant mice (Dominguez et al., 2007), but also the occurrence of *PTPRB* mutations in angiosarcomas (Behjati et al., 2014; Vicens and Posada, 2018) lends support for such a role. In a study towards hereditary factors that predispose to glioma development, using WES in a familial case, *PTPRB* appeared as one of the ten genes that displayed an accumulation of germline variants in the affected siblings (Backes et al., 2015). It remains to be investigated further whether and how the SNPs are indeed involved in specific pathways relevant for the development of brain tumors.

For quite some time also a potential involvement of *PTPRB* in the etiology of a multisystemic disease that includes intellectual disability, the so-called12q15 deletion syndrome, existed. Genetic mapping studies had narrowed down the involved region to less than one Mbp still harboring three genes, one being *PTPRB* (Alesi et al., 2017). Two recent studies that presented genetic data from novel cases, however, have brought an end to *PTPRB*’s candidacy and unambiguously point to gene *CNOT2* as the prime candidate for the 12q15 microdeletion syndrome (Alesi et al., 2019; Uehara et al., 2019). This does not mean that *PTPRB* is not involved in neuropathies, although its initial association with an increased risk for drug addiction (Ishiguro et al., 2008) has not been followed up as far as we know.

Genome-wide scans did also tie *PTPRB* to myopia, a rather common ocular disorder with a complex genetic component. Several different genomic loci have been linked to the disease and a few years back, by virtue of unique haplotypes in the Pennsylvania Amish founder population, also a novel significantly linked variant (HLOD = 3.77) in *PTPRB* could be added (Musolf et al., 2019). However, this SNP, with number rs2584021, represents a nonsynonymous variant (p.D57N) at a very conserved position within a Ricin B-type lectin domain in the longest RPTPβ isoform (isoform 3). An asparagine residue, however, is among the alternative residues observed at that position in homologous protein domains, as revealed by HOPE (Venselaar et al., 2010), thus further steps are needed to conclusively call the variant causal in myopia etiology. Another link with visual impairment came from exome sequencing studies on chronic central serous chorioretinopathy (cCSC) families (Schellevis et al., 2019). The *PTPRB* SNP rs61758735 (p.T1690I) did not only segregate in two unrelated families, it apparently also had been encountered in a previous large cCSC case-control study (Schellevis et al., 2018). Finally, by means of rs186466118 (p.S1376G) also *PTPRB* is listed as one of the candidate genes underlying familial Graves’ disease susceptibility (Hu et al., 2021). Both polymorphisms are within gene segments encoding fibronectin type III repeats (numbers 13 and 17 in isoform 1, respectively) and represent mutant residues normally not found at these conserved positions in this type of domain. Thus it is likely that these SNPs result in a molecular phenotype with respect to the extracellular interaction potential of RPTPβ.

Gene *PTPRH* encodes the enzyme SAP-1, which is particularly prominent in human intestinal epithelia. When compared to RPTPβ, SAP-1 has a considerably smaller extracellular segment, just eight FNIII domains, yet it shares substrates – including EGFR and IR - with its bigger subtype member (Shintani et al., 2015; Yao et al., 2017). Surprisingly, SAP-1 knockout mice had no morphological defects in their intestines and also the nutritional status of the animals appeared normal. SAP-1 deficiency, however, severely reduced the number of large, but not small, adenomas in *Apc* haploinsufficient animals (Sadakata et al., 2009), in line with an earlier cell model study that pointed to an inhibitory role in stomach cancer cell growth and motility (Noguchi et al., 2001) but arguing against a role for *Ptprh* in the initial transformation of intestinal cells. Crosses of the *Ptprh* knockout mice with inflammatory bowel disease animal models further demonstrated a supportive role for SAP-1 in intestinal immunity by fine-tuning the cytokine production in intestinal epithelial cells (Murata et al., 2015). Corroborating the absence of a clear phenotype in the SAP-1 deficient mice, no hereditary disease phenotype has been unequivocally associated with *PTPRH* variants. Whole exome sequencing in familial Parkinson disease cases, supplemented with functional studies and additional datasets, nominated *PTPRH* and four other genes as susceptibility gene candidates (Jansen et al., 2017), but this requires further investigation.

Gene *PTPRJ* was considered a suspect tumor suppressor early on, based on findings in cell models and mutant mice that collectively pointed to a role for the gene in vascular development and colon cancer susceptibility, and for the encoded protein (CD148, also known as density-enhanced phosphatase-1 or DEP-1) in the mechanism of cell contact growth inhibition (https://atlasgeneticsoncology.org/gene/41932/ptprj-(protein-tyrosine-phosphatase-receptor-type-j)). Studies probing its potential role in human hereditary cancer syndromes, however, did not meet up to the expectation. Initially, associations of *PTPRJ* SNPs with colorectal cancer (CRC) susceptibility were reported (Mita et al., 2010) but, as for *PTPN12*, systematic review of available data led to the conclusion that there is no significant association of *PTPRJ* variants with hereditary colorectal cancer (Belhadj et al., 2020). It may well be that other factors, such as additional low risk CRC alleles and/or gene-environment interactions, obscure its link to heritable CRC (Terradas et al., 2020). Since reports connecting *PTPRJ* variants to colon cancer risk continue to appear (Pelizzo et al., 2021) we may hope that meta-analyses in a perhaps far future could settle the issue.

*PTPRJ* is not only expressed in epithelial and endothelial cells; also hematopoietic linages contain the CD148 protein. Furthermore, detection of *PTPRJ* loss of heterozygosity in lymphomas also supported a tumor suppressor candidacy (Aya-Bonilla et al., 2013). Thus, the gene’s involvement in other cancer susceptibility syndromes has been studied as well. For example, *PTPRJ* variants were also tested for an association with the risk to develop breast, oesophagus, head and neck, lung and thyroid cancer. A meta-analysis of the various data obtained (Laczmanska and Sasiadek, 2019) fueled correspondence (Gholami and M MA, 2019) that in the end (Laczmanska and Sasiadek, 2020) led to the conclusion that the p.Q276P polymorphism is not associated with increased cancer risks and that the link for p.R326Q with colorectal cancer susceptibility is biased by data from a single study and thus awaiting independent confirmation. In fact this is echoed by results from a recent whole-exome sequencing project aimed at evaluating the clinical relevance of tumor suppressor gene variants, which illustrated the need for a careful classification of SNP effects (Balabanski et al., 2020). For example, rs1566734 in *PTPRJ* is listed as risk factor in SNP databases but its high minor allele frequency and its presence among centenarians rather points to a benign nature.

In mice *Ptprj* is required for proper heart development and vasculogenesis, providing the rationale to check for *PTPRJ* alterations that impact on heart and circulation diseases. Indeed copy-number variants for *PTPRJ* were found in two out of 316 congenital heart defect patients using a family trio-based study design (Sanchez-Castro et al., 2016). Also, the p.I1013S polymorphism in *PTPRJ* could be linked to mitral valve prolapse in one family, although segregation was not complete (Haskell et al., 2017). Kawasaki disease is an acute self-limited febrile vasculitis, mainly affecting young children and believed to be the product of a genetic susceptibility to incorrectly activate the immune system and an environmental trigger. Given CD148’s involvement in vasculogenesis and being also expressed in hematopoietic cells, in hindsight it seems logical that in a micro-array genotyping study (involving 164,395 SNPs, 119 Polish patients and over 6K controls) the polymorphism rs151078858 in *PTPRJ* was among the five that were most statistically linked with Kawasaki disease (Buda et al., 2021). Moreover, two protein-truncating *PTPRJ* alleles (p.T38Pfs9* and p.S626Afs7*) have recently been discovered in a study towards genes underlying inherited thrombocytopenia and, importantly, functional studies in zebrafish and mouse models underscored the important role for *PTPRJ* in platelet biogenesis (Senis et al., 2009; Marconi et al., 2019; Nagy et al., 2020). A decade ago already, three *PTPRJ* polymorphisms (rs1566734, rs1503185 and rs4752904) were linked to human platelet reactivity and suggested to lower the risk of heparin-induced thrombocytopenia (Rollin et al., 2012) although this could not be confirmed in a French patient cohort (Lioger et al., 2016). Obviously, there is still a lot to discover for inherited platelet disorders (Pluthero and Kahr, 2019).

As said, *PTPRO* is firmly expressed in brain and kidney but the encoded protein, GLEPP1 (glomerular epithelial protein 1), is also found in bone (Shalev and Elson, 2019) and other tissues. Expression of this receptor tyrosine phosphatase in podocytes provides a rationale why *PTPRO* mutations are causative of childhood-onset nephrotic syndrome (Ozaltin et al., 2011). Detailed electron microscopic studies on nephrotic material from GLEPP1 deficient mice and other glomerular disease models, including Alport syndrome, revealed that podocyte invasion into the glomerular basement membrane (GBM) preceded GBM thickness alteration and a gradual loss of podocyte foot processes during disease progression (Randles et al., 2016). Nephron functionality apparently is very vulnerable because many different podocyte-related genes have been uncovered as monogenetic cause of nephrotic syndromes, and mutant *PTPRO* alleles are regularly detected (Trautmann et al., 2018; Thakor et al., 2021), although this could be population dependent (Al-Hamed et al., 2013).

*PTPRO* alleles have also been associated with the risk for acute renal graft rejection (Ghisdal et al., 2017; Cargnin et al., 2020) but mechanistically this calls upon a different function for the gene. The gene is also expressed in B cells but there it gives rise to the shorter isoform PTPROt, due to alternative promoter use, that is essential for B-cell proliferation and B-cell signaling *via* the kinases Lyn and ZAP-70 (Motiwala et al., 2010). *PTPRO*’s impact on B cell proliferation is supported by aberrant expression in B-cell chronic lymphocytic leukemias and by the detection of a shared germline *PTPRO* variant (rs6175411) in a pair of monozygotic twins with hematological pre-malignancies (Hansen et al., 2015). Additionally, in one of the first WGS studies addressing hereditary factors predisposing for chronic obstructive pulmonary disease (COPD) this *PTPRO* gene variant rs61754411 came out as number one association across the exome (*p* = 4.0 × 10^–5^), although genome-wide significance was not reached. Further studies revealed that this rare nonsynonymous variant (p.N370K, in the fourth FNIII domain of GLEPP1) attenuated EGFR signaling in response to several stimuli in primary epithelial cells (Radder et al., 2017), which fits with earlier reports on a link between the EGFR pathway and chronic lung diseases (Vallath et al., 2014). Although early days, the above results call for a detailed characterization of *PTPRO* genotype-phenotype correlations.

As indicated at the start of this section, gene *PTPRQ* is the odd-one-out in this receptor-type PTP subclass (Chicote et al., 2017a); it encodes one of the few classical PTPs that have phosphoinositides rather than phosphotyrosine-containing proteins as substrates (Pulido et al., 2013). Early this century PTPRQ was identified as the 275 kDa hair-cell antigen in the inner-ear (Goodyear et al., 2003). Knockout mouse studies demonstrated that PTPRQ is an essential component of the stereocilia hair-bundle shaft connectors since deficiency results in hearing loss (Goodyear et al., 2003). In 2010 the formal proof was published that inactivating mutations in the gene are also responsible for deafness in humans (Schraders et al., 2010; Shahin et al., 2010) and many more have followed since (Gao et al., 2015; Sang et al., 2015; Eisenberger et al., 2018; Wu et al., 2018; Ozieblo et al., 2019; Sang et al., 2019; Safka Brozkova et al., 2020; Chen et al., 2021a; Mahmood et al., 2021; Yang et al., 2021). For a more detailed description of the genetic data we refer to excellent recent reviews (Kremer, 2019; Richardson and Petit, 2019). Currently it is still unclear whether it is PTPRQ’s main task to provide a cell surface coat at the stereocilia base in hair cells, hence to take up a structural role, or that it is supposed to perform an enzymatic role to support and maintain cochlear functionality. In the mouse model, initially the hair-cell stereocilia are held together despite absent shaft connectors but postnatally the hair-bundles gradually deteriorate and cells die (Goodyear et al., 2003), which could lend support to both viewpoints. The collection of disease-causing mutants in human is also ambiguous. Several disease alleles represent point mutations that affect the extracellular portion of the molecule, which may support a cell adhesive role. In contrast, the p.W2294* variant only lacks the last six amino acids of the protein and apparently acts as dominant negative (reviewed in (Kremer, 2019; Richardson and Petit, 2019)) supporting an essential task for the intracellular segment as lipid phosphatase or as interaction platform for other proteins. Of note, the very C-terminus in PTPRQ represents a PDZ domain binding site (Barnea et al., 2016), providing a potential regulatory mechanism (Hendriks et al., 2018). A confounding factor is that the PTPRQ primary sequence may picture a receptor-like inositol lipid phosphatase but that it is still enigmatic whether and how its enzymatic activity is controlled. Thus, also structural changes on the outside may well impact the protein’s intracellular role, and *vice versa*. Unfortunately, the inner ear is one of the hardest tissues to model and modify, and structure-function studies thus remain dependent of cumbersome studies in accessible model systems like zebrafish. It should be noted that, although *PTPRQ* is also expressed in many other cilia-bearing cells, patients with *PTPRQ* disease variants thus far only present with deafness and vestibular dysfunction, pointing to functional redundancy in the unaffected tissues. It may be relevant therefore to mention that an evolutionary relationship between PTPRQ and the tetraspanin-associated uroplakins family has been proposed (Chicote et al., 2017b), although Ockham’s razor rather urges us to point to the other R3-type RPTP subfamily members (Chicote et al., 2017a) or to the dozen or so non-classical PTPs that demonstrated phospholipid phosphatase activity (Pulido et al., 2013).

### 2.13 *PTPRC*

The primary structures of CD45 protein isoforms RA, RB and RC, which result from alternative spliced transcripts of the *PTPRC* gene, were known well before it was realized that their intracellular tandem repeated sequences in fact represent two PTP domains (Figure 1). The membrane-proximal PTP domain harbors enzymatic activity, regulated *via* intermolecular homodimerization, and the more membrane-distal one is suspected to fine-tune activity and/or substrate selectivity of the first. Mouse studies revealed that CD45 deficiency as well as hyperactivity have major consequences for cells of the hematopoietic lineage, and also the decades of studies on human materials established *PTPRC* as an important immunomodulatory gene with impact on autoimmune and infectious diseases (reviewed in (Tchilian and Beverley, 2006; Al Barashdi et al., 2021)). Inactivating mutations in *PTPRC*, for example, are responsible for some autosomal recessive cases of severe combined immunodeficiency (SCID), and SNP rs17612,648 confers a susceptibility risk for viral infections. The latter polymorphism actually represents a synonymous point mutation (p.P59=) in exon four of the *PTPRC* gene that affects a splice silencer region; the C>G change enhances inclusion of exon 4, altering the CD45 splice forms displayed by hematopoietic cells. As was also confirmed in a SNP genocopy mouse model, the net result is an increase in primed and effector memory T-cells, in the activation of Lck and in proliferation (Dawes et al., 2006). For another SNP in *PTPRC*, rs10919563 that resides within an intron further downstream in the gene, it is as yet unclear how this mechanistically may impact on immune cell functioning, but multiple reports have hinted at an association with autoimmune diseases such as familial rheumatoid arthritis, systemic lupus erythematosus and primary Sjögren’s syndrome (Tchilian and Beverley, 2006; Al Barashdi et al., 2021). Importantly, this rs10919563 SNP proved instrumental in predicting the responsiveness to anti-TNF therapy, with G>A allele carriers showing a poor response to anti-TNF therapy ((Lee and Bae, 2016) and references therein). However, in a more recent study (Gibson et al., 2021) rs10919563s predictive power did not prevail. Larger studies will be needed to unequivocally determine whether this *PTPRC* SNP is of help in predicting responders for this high cost biologic treatment.

### 2.14 *PTPRD, PTPRF* and *PTPRS*

The type IIA subfamily of receptor-type PTPs harbors three members, RPTPδ, LAR and RPTPσ, that are encoded by genes *PTPRD*, *PTPRF* and *PTPRS*, respectively. These proteins all have similar extracellular segments consisting of three sequential immunoglobulin-like domains followed by usually eight fibronectin type III repeats, a segmental combination that is reminiscent of cell-cell adhesion molecules. Their intracellular portions harbor twin PTP domains of which the membrane-proximal one is the catalytically active moiety (Figure 1). Expression patterns for the three genes are distinct but certainly display overlap and the same can be said about their ligand and substrate specificity. A thorough review with a specific focus on their role in the brain was published recently (Cornejo et al., 2021). A decade ago, only limited evidence linking these three RPTP genes to hereditary diseases had been gathered (Hendriks and Pulido, 2013). Meanwhile, the tremendous progress in DNA sequence analyses techniques generated an explosion of genetic associations but for the largest part these only implicate gene *PTPRD*. Realizing that *PTPRD’s* size (some 2.3 Mb and spanning at least six other transcription units) is 15–20 times that of *PTPRF* and *PTPRS*, it is perhaps to be expected that *PTPRD* covers more associations that result from GWAS and other analyses (see https://www.ebi.ac.uk/gwas/genes/PTPRD). In the following, we will therefore try to filter hypes and hopes, and refer to a review by Uhl and Martinez (Uhl and Martinez, 2019) for further details on *PTPRD*’s contribution to brain diseases.

*PTPRD* polymorphisms have been associated with susceptibilities to cancer, notably renal cell carcinoma (rs2279776; p.G1418= (Du et al., 2013)) and endometrial cancer (rs2475335; intron variant (Painter et al., 2018)). *In vitro* studies had pointed to STAT3 as being an important RPTPδ substrate (Veeriah et al., 2009). Combined with germline *PTPRD* mutations, among which a p.W775* nonsense variant, that were observed in Ewing sarcoma cases (Jiang et al., 2013)—a tumor type often displaying elevated STAT3 activity – this lends support for an important role of RPTPδ in dephosphorylation of STAT3, downstream of insulin growth factor receptor (IGF-1R). Indeed, two-thirds of the patients with germline RPTPδ mutations responded well when treated with anti-IGF-1R antibodies suggesting that *PTPRD* status may have implications for therapy (Jiang et al., 2013). Also, a *PTPRD* germline variant (p.C1428G) was identified exclusively in smoker patients among Brazilian non-small-cell lung cancer patients (Couto et al., 2017) and a partial deletion (9p23 (9101605_9521604)x1) in *PTPRD* is suspected to have predisposed a child for developing glioblastoma (Gambale et al., 2019), but clearly more data is needed to corroborate the gene’s candidacy as a cancer susceptibility gene.

First ‘disease ties’ for *PTPRD* in fact addressed restless legs syndrome (RLS) or Willis-Ekbom disease, an autosomal dominant disorder that causes insomnia due to an irresistible desire to move the legs. Dopaminergic agonists are used to treat affected individuals but dopaminergic transmission-related genes are not among the identified predisposing genomic loci. Rather, processes that contribute to spinal cord interneuron development, limb development, and iron metabolism appear to be affected. Using primarily intronic SNPs, *PTPRD* emerged as one of the strongest genetic factors in the risk to develop RLS ((Jimenez-Jimenez et al., 2018) and references therein; *p* < 10^–8^). On a previous occasion (Hendriks and Pulido, 2013) we proposed, based on findings in *Ptprd* mutant mice (Uetani et al., 2006), that alterations in RPTPδ mRNA levels may impact on motor neuron axon guidance during limb development and on trans-synaptic signaling. A few years later further proof in support of such a mechanism came from additional studies in mice and RPTPδ mRNA and SNP measurements in human *postmortem* brain samples (Drgonova et al., 2015). Since all six *PTPRD* polymorphisms that were included in the study (rs2381970, rs4626664, rs197519, rs7470838, rs2296094 and rs10115782; with nominal p values ranging from .002 to .05) represent intronic variants, this *PTPRD*-RLS association provides a strong argument to include level-of-expression variation as a phenotypic consequence in discussions on the relevance of SNPs that reside outside protein-coding regions. This notion is further supported by the finding that in another study several RPTPδ missense variants (p.Q447E, p.T781A, and p.R995C) did not co-segregate with RLS (Gan-Or et al., 2015a). The net effect of these missense variants on RPTPδ protein level and activity, however, remains to be elucidated. And one should not forget that several other genes reside within its boundaries that may be affected by the SNPs as well. Among individuals experiencing migraines (Fuh et al., 2016) or suffering from Parkinson disease (Gan-Or et al., 2015b) *PTPRD* intronic SNPs did not significantly associate with RLS, leaving ample space for other RLS risk-associated genes to contribute under these two disease conditions.

Gene *PTPRD* has also been linked to addiction-related phenotypes but the modest association signals could also be interpreted as being nearby the responsible loci (Drgonova et al., 2015). Irrespective, using a cocaine reward set-up for heterozygous *Ptprd* knockout mice and wildtype controls, Uhl and others (Uhl et al., 2018) noted that decreased RPTPδ levels are paralleled by reduced self-administration. Moreover, *in vivo* administration to wildtype mice of an inhibitor that has higher affinity for RPTPδ than for the homologous RPTPσ led to a similar effect, pointing to *PTPRD* as a potential anti-addiction therapeutic target (Uhl et al., 2018). Other studies also link *PTPRD* with behavioral or neurological phenotypes. For example, a study in young Korean women that employed a ‘five-factor model of personality’ revealed an association with the ‘Openness domain’ in the model (Kim et al., 2013). The reported SNPs (rs2146180, rs10976737 and rs7861684) reside just downstream of the PTPRD transcript region, leaving any mechanistic insight open for speculations involving gene regulatory elements. A subsequent meta-analysis of genetic data on personality in Korean cohorts (Kim et al., 2015) rather linked *PTPRD* (by means of rs1029089, again flanking the 3′ end of the gene) to the ‘Agreeableness domain’, and this result could be confirmed in twins. Also investigations towards the risk for autism spectrum disorder (ASD) in Japanese and Han Chinese populations have yielded *PTPRD* as being associated (*p* = 5.3 × 10^–6^), again using a SNP (rs7875560) downstream of the gene (Liu et al., 2016). A meta-analysis of two large GWAS studies focusing on obsessive-compulsive disorder (OCD) did not result in SNPs with genome-wide significance but it did have variants near *PTPRD* (*p* = 4.1 × 10^–7^) amongst the top signals (International Obsessive Compulsive Disorder Foundation Genetics Collaborative and OCD Collaborative Genetics Association Studies, 2018). To add to the complexity, a search for genetic components contributing to ‘Social conformity’ in a Chinese cohort, including twin datasets, also yielded a strong association (*p* = 4.8 × 10^–6^), this time using the *PTPRD* intronic SNP rs2381801 (Chen et al., 2018a). Collectively, these data again indicate the need to assess mechanistic effects at the transcript, cellular and tissue level for SNPs residing outside protein-coding genomic regions. In a study for genetic factors contributing to Alzheimer’s disease etiology, notably neurofibrillary tangles disposition, this was indeed performed. Intronic *PTPRD* variant rs560380 associated with neurofibrillary tangle counts but not with other neuropathologic traits, and despite extensive research in *postmortem* materials and cell lines, there is no sign that rs560380 influences RPTPδ mRNA expression (Chibnik et al., 2018).

Continuing on the line that RPTPδ acts in neural development and functioning, hereditary forms of intellectual disabilities come to mind as novel territory for *PTPRD* etiological involvement. Targeted next generation sequencing (NGS) including over four hundred intellectual disability/developmental delay-related genes and hundred and twelve patients indeed supported *PTPRD* involvement; a p.S1845Rfs*2 variant was found in one case (Yan et al., 2019). A method termed genetic evolved random forest (GERF) also yielded evidence linking the gene to mild cognitive impairment (Bi et al., 2021). Underscoring the importance of proper RPTPδ transcript and protein levels for normal brain development and function it is of note that also copy number variations (CNVs) for *PTPRD* have been detected in patients with complex neurodevelopmental disorders (Servetti et al., 2021), including Dandy-Walker malformations (Schumann et al., 2016). The microdeletions involving *PTPRD* have been taken up as part of a so-called “BGNADP” motif, comprising gene *BTD*, *GALNT10*, *NMUR2*, *AUTS2*, *DLG2* and *PTPRD*, that would signify a key network determining intellectual disabilities and developmental delay (Gao et al., 2018). Based on the above findings it is tempting to suggest that also the *PTPRD* intronic variant rs35331017 that was associated with the risk for spontaneous preterm birth following maternal stress (Hong et al., 2021) is impacting on the gene’s transcript levels.

*PTPRD’s* candidacy as risk gene was also brought up in the context of other developmental defects. Disease links with normal hearing function (Girotto et al., 2014) and with modic change, a form of lumbar disc degeneration that contributes to disabling low back pain (Freidin et al., 2019) have penetrated the literature. Some insight on how RPTPδ may influence a process like osteogenesis may ultimately come from investigations on the interplay of genetic and environmental factors that determine bone mineral density. Recent studies point to a protective role of uric acid against bone loss, and an interaction effect of serum urate levels and rs10977015 in *PTPRD* on bone mineral density was suggested using data from the United Kingdom Biobank cohort (Yao et al., 2021). Similarly, in a search for genetic components that may associate with circulating glycine levels in the risk to develop coronary artery disease in women, *PTPRD* was among the twelve loci that linked to glycine metabolism but the latter could not be tied to coronary artery disease risk (Jia et al., 2019). Such a putative regulatory role in the metabolic pathway leading from choline to urea may, of course, be a rather indirect effect of RPTPδ signaling but it may also build a broader portfolio of *PTPRD* as metabolism regulator gene. The *PTPRD* p.R995C variant (rs35929428), for example, was found to associate with the risk to develop non-alcoholic fatty liver disease (*p* = .015, odds ratio = 5.00) and this may well be due to enhanced phospho-STAT3 levels and consequent hepatic lipid accumulation and fibrosis (Nakajima et al., 2018). Perhaps *PTPRD*’s candidacy to genetically link (*p* = 1.3 × 10^–6^) to the chance of developing resistant hypertension, defined as suffering from uncontrolled blood pressure despite the use of maximum tolerated doses of multiple antihypertensive medications, should be viewed in a metabolic context as well (El Rouby et al., 2019). Along those lines, the p.T2071I RPTPδ mutant that was identified *via* WES in one of six trios is being considered as pathogenic in maturity-onset diabetes of the young (Shim et al., 2015). Also, using mouse models and methylation-specific PCR analyses, the hypermethylation -hence silencing- of *PTPRD* correlated with decreased insulin receptor signaling and type 2 diabetes susceptibility (Chen et al., 2015). Furthermore, a type 2 diabetes-related CNV (nsv8414) with marginal significance was detected for *PTPRD* (Yan et al., 2018) and intronic SNP rs17584,499 in the gene significantly associated (*p* = 8.5×10^−10^) with the incidence of this disease in the Chinese population (Tsai et al., 2010; Chen et al., 2021b).

Having come to know *PTPRD* as a prime suspect for a plethora of disease conditions it is rather contrasting to see the limited hereditary dangers that have been tied to gene *PTPRS*, at least for the time being. Again largely thanks to studies in mutant mouse models the protein encoded by *PTPRS*, RPTPσ, demonstrated important roles in development and function of the nervous system (Cornejo et al., 2021), including the control of synaptic transmission (Brown et al., 2020). Also, RPTPσ functionality turned out to be a two-edged sword in the fight against intestinal inflammatory processes (Ohtake et al., 2018); the protein not only protects the permeability of the epithelial layer (Murchie et al., 2014) but also keeps dendritic cells under control (Bunin et al., 2015). Collectively, this corroborates the association of SNPs rs886936, rs17130, and rs8100586 in *PTPRS* –all influencing the inclusion of RPTPσ’s third Ig-like domain–with the risk to develop ulcerative colitis (Muise et al., 2007). Three other SNPs more downstream in *PTPRS* (rs1143699, rs4807015, and rs1978237) were found to confer a risk to develop type 2 diabetes in a Swedish cohort (Langberg et al., 2007), which suggests the possibility of isoform-specific pathological ties for the gene. Quite recently, based on two additional SNPs that encode missense variants, *PTPRS* has also been linked to nonsyndromic cleft palate (Hoebel et al., 2017) but these predictions need further proof.

The third gene in this PTP receptor-type subclass is *PTPRF*, which encodes LAR (for leukocyte common antigen-related protein). *Ptprf* phosphatase deficient mice displayed neuronal functional deficits (Van Lieshout et al., 2001; Xie et al., 2001; Dunah et al., 2005) and defunct mammary gland development, albeit with variable penetrance (Schaapveld et al., 1997). Overexpression studies pointed to a role for LAR in dephosphorylating the insulin receptor and, in line, one report linking a genetic *PTPRF* promoter variant to obesity and insulin resistance appeared in the literature (Miscio et al., 2004). Insulin resistance may lead to coronary artery disease, and in two studies indeed the intronic polymorphism rs2782641 in *PTPRF* was found to associate with the disease as a recessive trait in type 2 diabetic patients (Wellcome Trust Case Control Consortium, 2007; Menzaghi et al., 2008). Further reports on germline mutations since then rather point to the original mammary gland phenotype in the knockout mice than towards metabolism or brain development. The first report was on an inherited reciprocal balanced translocation involving *PTPRF* that was detected in a syndromic amastia patient (Ausavarat et al., 2011). Amastia is an extremely rare genetic disorder that results from the lack of mammary ridge development *in utero* leading to full absence of breasts. A further corroboration came from the notion that within an extended consanguineous family the individuals that suffered from athelia, a developmental abnormality that is defined by absence of the nipple-areola complex, were all homozygous for a frameshift mutation (p.V616Efs*49; rs1131692054) in *PTPRF* (Borck et al., 2014). Occurrence of athelia is very rare and it may also present as part of more complex pathologies, including Kabuki syndrome. Still, dozens of cases that involve athelia and require molecular underpinning are known (Ausavarat et al., 2011; Baldridge et al., 2020), and some of these may shed further light on the mechanism how *PTPRF* mutations actually lead to this developmental anomaly. This will be instrumental in including LAR’s recently proposed connection with nonmedullary thyroid cancer susceptibility (Zhu et al., 2019).

### 2.15 *PTPRG* and *PTPRZ1*

The R5 subgroup of RPTPs contains two members that clearly separate from the other PTPs by virtue of their unique extracellular domain that is heavily glycosylated and N-terminally start with first a carbonic anhydrase domain that is followed by a single fibronectin type III repeat (Figure 1). In fact, the enzyme encoded by PTPRZ1 (RPTPζ, formerly also confusingly termed RPTPβ) was the first receptor-type PTP for which corresponding ligand molecules (Peles et al., 1995) as well as their effect on RPTP enzyme activity (Fukada et al., 2006) was documented. The *PTPRG* and *PTPRZ1* genes both encode multiple isoforms and these even include secreted, soluble ‘decoy receptor’ or ‘ligand-type’ extracellular variants (Shintani et al., 1997; Fujikawa et al., 2017). The expression patterns for both genes, however, are quite distinct, with *PTPRG* being rather widely expressed whereas *PTPRZ1* transcripts appear limited to the nervous system.

The chromosomal location of *PTPRG*, 3p14-21, is in a gene-dense area that is frequently deleted in specific tumors and that is also linked to intellectual disabilities known as 3p deletion syndrome. *PTPRG*, therefore, represented an appealing tumor suppressor gene candidate and research over the years yielded many different ways by which the function of RPTPγ, its encoded protein, can be impaired. Occasionally deletions, but mostly missense mutations and transcriptional silencing by means of interfering RNAs (notably the long non-coding RNA transcribed from *PTPRG-AS1* that overlaps with the last exons in *PTPRG*) or promoter hypermethylation were found, and a comprehensive review of *PTPRG*’s role in cancer has recently been published (Boni and Sorio, 2021). In addition, an extensive overview of non-cancerous disease links for *PTPRG* appeared in the literature (Boni et al., 2022), and we therefore here limit ourselves to the main findings. A report that actually merges both hereditary diseases and cancer deals with a case of infantile myofibromatosis (Linhares et al., 2014), a disorder that is characterized by benign tumors in various tissues, notably striated muscles. One of the causative genes for the disorder is *PDGFRB* (Guerit et al., 2021), encoding the RPTPγ substrate PDGF receptor beta. In one family, however, surprisingly a limited penetrance by the *PDGFRB* disease allele was observed. It turned out that affected family members additionally carried a p.V426M missense mutation in *PTPRG*, suggesting an additive effect of mutations in both the substrate and the phosphatase (Linhares et al., 2014).

In a recent study (Hansen et al., 2020) Hansen and others demonstrate that in transgenic mice the carbonic anhydrase domain in RPTPγ serves as an HCO_3_^−^ sensor on endothelial cells, thereby regulating microvascular perfusion and blood pressure upon metabolic acid-base changes. Furthermore, they note an association for predicted loss-of-function variants in *PTPRG* with human ischemic vascular diseases using United Kingdom Biobank data. This lends support to an earlier finding on ischemic stroke incidence in African-Americans that yielded a nominal association (*p* < 10^–6^) for *PTPRG* intron variant rs704341 (Carty et al., 2015). It may also provide a mechanistic cue for the strong association (*p* = 1.3 × 10^–6^) of *PTPRG* variants with Fuchs’ endothelial corneal dystrophy, a disorder causing the gradual loss of corneal endothelial cells (Baratz et al., 2010; Wang et al., 2014b; Lau et al., 2014). Genome-wide significance was reached (*p* = 3.98 × 10^–8^), however, for the association of rs7609954 in *PTPRG* and Alzheimer’s disease (Herold et al., 2016). Finally, combining a WES analysis of fourteen complete parent–offspring trios with sporadic schizophrenia, which yielded one case with a disruptive *PTPRG* mutation, and the identification of five additional mutant alleles *via* targeted sequencing in an independent cohort of 48 patients also linked *PTPRG* to schizophrenia (Kranz et al., 2015; Kranz et al., 2016; Cressant et al., 2017). The latter two disease associations are shared with its paralogue, *PTPRZ1* (Nagai et al., 2022).

*PTPRZ1* SNPs significantly associating with the risk to develop schizophrenia have been reported (Buxbaum et al., 2008) but the gene’s link with Alzheimer’s Disease thus far is based on expression levels of RPTPζ isoforms and of its ligand pleiotrophin in *postmortem* material (Zhao et al., 2021). Because schizophrenia-associated genes represent promising candidates for predicting antidepressant efficacy, Su and others have tested five *PTPRZ1* SNPs for an association with anxiety remission status in two Chinese cohorts that were stratified for the medication received, but correction for multiple testing aborted the potential associations (Su et al., 2021a). However, also in view of the recent compelling data that come from multiple behavioral studies in mice (Cressant et al., 2017; Fernandez-Calle et al., 2018; Fujikawa et al., 2019; Tanga et al., 2019) it is a matter of time to be able to pinpoint *PTPRZ1* variants as components in the multifactorial central nervous system disorders. Studies involving *Ptprz1* mutant mice and various cell models have established a regulatory role in neuroinflammation and (re)myelination (Fujikawa and Noda, 2016; Fernandez-Calle et al., 2020; Nagai et al., 2022), and thus multiple sclerosis as disease link also comes to mind. This is, however, as yet not supported by human *PTPRZ1* mutations associating with this chronic inflammatory demyelinating disorder. Not only (micro) glial cells but also B-cells express RPTPζ, expanding its modulatory effect on immune cell survival (Cohen et al., 2012). Perhaps in this light one should interpret the reported candidacy of *PTPRZ1* as a risk gene to develop pneumoconiosis, dust-triggered irreversible fibrosis of lung tissue (Wang et al., 2015). Causative genes for the autosomal recessive disease termed hyperlysinemia, defective lysine degradation, largely remain to be discovered and a gene, encoding α-aminoadipic semialdehyde synthase, just downstream of *PTPRZ1* has been listed as one of the culprits. Interestingly, in two patients the involved deletion also affected *PTPRZ1* and this was paralleled by a more severe neurological phenotype in the individuals (Houten et al., 2013). Details on how the *PTPRZ1* co-deletion impacts on lysine catabolism remain to be elucidated.

### 2.16 *PTPRK, PTPRM, PTPRT* and *PTPRU*

The four genes *PTPRK*, *PTPRM*, *PTPRT* and *PTPRU* encode receptor-type PTPs that have an extracellular portion that is again reminiscent of cell adhesion transmembrane molecules; a single Ig-like segment and a small series of FN-III domains. As a more clearly discriminating feature, however, the encoded proteins (RPTPκ, RPTPμ, RPTPρ and PCP-2, respectively) all have an N-terminal MAM (Meprin, A5 neuropilin, RPTPµ) domain (Figure 1). RPTPµ was the first to be cloned and subsequently used, based on its cell adhesion molecule-like appearance, in aggregation and functional studies. These revealed that the MAM-bearing RPTPs are exclusively homophilic *trans* cell-cell adhesion receptors that contribute to the regulation of cadherin-based adhesion junctions and convey their signals to catenin proteins and other important signaling mediators, including Akt and STAT3 (Craig and Brady-Kalnay, 2015; Kim et al., 2018). In line, *PTPRM* and *PTPRT* make part of the list of genes that are found deleted in tumors of the digestive tract (Laczmanska et al., 2014).

Limiting ourselves to connections with hereditary diseases only, gene *PTPRK* brings in an interesting case. First reports were on an association with the autism spectrum of disorders (O'Roak et al., 2012a; O'Roak et al., 2012b) and its link with neuropathologies was later on extended to include Alzheimer’s disease (Chen et al., 2018b). It should be noted, however, that *PTPRK* is directly upstream of gene *THEMIS* (thymocyte-expressed molecule involved in selection), and rats that suffer from a co-deletion of both genes display defective T-cell maturation because of T-helper immunodeficiency (Iwata et al., 2010). In humans, the locus is under investigation because of SNP associations with the risk to develop coeliac disease (Bondar et al., 2014; Senapati et al., 2015) and a potential protective effect against multiple sclerosis (Davies et al., 2016) and type 1 diabetes (Inshaw et al., 2018). Intriguingly, rescue experiments in the aforementioned mutant rats underscored that the RPTPκ and Themis proteins jointly contribute to the immunodeficiency phenotype (Iwata et al., 2010), providing a real conundrum as to what the net effects are of the many variants that reside in intronic regions and the intergenic sequence in the *PTPRK/THEMIS* locus. Adding to the puzzle, in the ClinVar database *PTPRK* mutations have been associated with hereditary breast and ovarian cancer (p.R1398Q; VCV000981859) and with metastasis from primary bronchial carcinoid tumor (VCV000916695) but these may also affect PTPRK-AS1, the antisense transcript that is derived from the complementary strand.

Gene *PTPRM*, encoding RPTPµ, is expressed in many different cell types, including the arterial endothelium, and deletion studies in mice yielded viable and fertile mice that show a mild arterial dilation defect in support of a mechanotransductory role for this transmembrane PTP (Koop et al., 2005). In humans, however, the currently reported associations with hereditable disease states point to neurological and, perhaps, autoimmune-related issues. To start with the latter, an early report on rs4798571 associating with multiple sclerosis could not be replicated (Varade et al., 2012) but a study aimed at identifying genetic factors influencing interferon alpha serum levels in systemic lupus erythematosus patients pointed to rs930926 (Ghodke-Puranik et al., 2020). However, this SNP is 100 kbp downstream of the *PTPRM* gene, in the midst of several pseudogenes (including, interestingly, one of *THEMIS*). The report on a detection of a *de novo* 1.1-Mbp duplication, involving a genomic region harboring three genes among which *PTPRM*, in a patient with hemiplegic cerebral palsy (Zarrei et al., 2018) actually comes close to the arterial phenotype noted in *Ptprm* knockout mice because this disease is characterized by one-sided defective posture and movement that is due to a vascular insult, a venous infarction, or brain malformation. The option that *PTPRM* may impact on brain development and function is further supported by its association with the risk to develop medulloblastoma (Dahlin et al., 2020). The gene was also included in a study on variants associated with schizophrenia but effectively *PTPRM* was found deleted once in the case group and also once in controls (Kushima et al., 2017).

The first PTP superfamily-focused screen for mutations in cancer specimens highlighted frequent alterations in six classical PTP genes, with *PTPRT* being the number one hit (Wang et al., 2004) putting a tumor suppressor function for the encoded RPTPρ in the limelight. In line, observed mutations in the extracellular and intracellular portion of the protein were demonstrated to affect cell-cell adhesion and proliferation, respectively. Moreover, studies in mice, including gene knock-out and transposon hopping screens, also corroborated RPTPρ as tumor suppressive and molecular studies highlighted paxillin and STAT3 as relevant substrates (reviewed in (Scott and Wang, 2011)). Another report, however, tones down the frequency and importance of *PTPRT* alterations in sporadic human cancers (Lee et al., 2007). Switching gears to hereditary *PTPRT* variants, again a link with neoplastic pathologies was found. For example, in addition to *PTPRD*, also *PTPRT* mutations associate with a better outcome for non-small cell lung cancer patients that receive immune checkpoint inhibitors (Wang et al., 2021). Also, the risk to develop esophageal squamous cell carcinoma appears sensitive to an SNP in *PTPRT*’s last exon that, interestingly, corresponds with a miR-218 binding site in its mRNA (Yao et al., 2015). *PTPRT* resides in a genomic region that is often deleted in myeloid disorders but deletion variants are occasionally found in lymphoproliferative diseases such as myeloma as well (Mitev, 2021). This deletion of *PTPRT* had also been noted as representing one of the thirteen hotspots that were detected while comparing parent-parent-child DNA samples in an Attention Deficit Hyperactivity Disorder study (Bradley et al., 2010) and mouse mutants lacking RPTPρ activity indeed show altered behavior (Thirtamara Rajamani et al., 2015). Further associations for the gene with neurological pathologies come from the genetic analysis of families with intellectual disability cases (Schuurs-Hoeijmakers et al., 2013) and the detection of a compound heterozygote mutation for *PTPRT* in one proband amongst a cohort of families with congenital brain malformations and/or intellectual disability cases (Karaca et al., 2015). Furthermore, intronic variant rs6030462 in *PTPRT* appeared as risk factor in sporadic Parkinson’s disease and amyotrophic lateral sclerosis in a Chinese cohort (Lu et al., 2021). Yet another intronic *PTPRT* variant (rs490514), again in the Chinese population, associated with congenital heart disease susceptibility (Lin et al., 2015). These brain and heart connections for *PTPRT* intronic variants may provide some background to the finding that transcript level-influencing SNPs in the gene were detected in a genome-wide meta-analysis of the genetics of gait speed in older persons (Ben-Avraham et al., 2017). The SNPs did not reach genome-wide significance yet could be highlighted as suggestive significant associations (*p* < .0001) given their low recombination rate and linkage disequilibrium. Knowing that RPTPρ is expressed in cells of the immune system as well, associations with immune-related disorders were to be expected. Indeed, an early report suggested an involvement in the risk to develop rheumatoid arthritis (Julia et al., 2008); genome-wide significance was not reached (*p* = 3.8 × 10^–6^ for rs11086843) but in a replication study *PTPRT* SNP rs2476601 showed a good correlation with the size of the genetic effect. More recently, PTPRT polymorphisms were also connected (*p* = 9.7 × 10^–8^) with a potential protective role in malaria (Milet et al., 2019) and with an influence on the effectiveness of glatiramer acetate in the treatment of multiple sclerosis (Zarzuelo-Romero et al., 2021). Altogether, the many suggestive links for this RPTP gene urge for a lot of follow-up studies.

The last member of the MAM domain-containing RPTP genes, *PTPRU*, also represents an interesting case. Directly following its initial cloning and also in subsequent papers the membrane-proximal PTP domain in the encoded protein (PCP-2) was viewed as *bona fide* phosphatase (Wang et al., 1996; Yan et al., 2006) but quite recently PCP-2 was rather shown to be a pseudophosphatase that, by sequestering but not modifying phosphotyrosine-containing substrates, acts as a protector in competition with active superfamily members (Hay et al., 2020). Results were obtained *in vitro* and in modified cell lines, and future studies on the *in vivo* role of *PTPRU* and its gene variants might reveal how this substrate-sequestering scenario helps in explaining the findings. Thus far, studies in chicken, zebrafish and mice have pointed to a role for PCP-2 in embryonic development, notably somitogenesis, cardiogenesis, and formation of neurogenic and sensory organs, by impacting on Hippo, Notch and Wnt signaling pathways (Aerne et al., 2003; Aerne and Ish-Horowicz, 2004; Gu et al., 2019; Grad et al., 2022). Clear links to developmental diseases, however, have not emerged yet. There is the mentioning of rs10914351, an SNP around 240 kbp downstream of *PTPRU*, that associates with sleep duration (Ollila et al., 2014), and two *PTPRU* missense mutations that appear linked to short stature have been deposited in ClinVar (rs559788899, p.P844L; rs540351799, p.M1389L). In the literature, the verdict is still out whether *PTPRU* represents an oncogenic or a tumor suppressor gene (Craig and Brady-Kalnay, 2015) but from the limited studies the picture emerges that PCP-2 expression levels are critically determining its scavenger/protector efficacy in the competition with enzymatically active PTP family members for tyrosine phosphorylated substrates, and that its transcript levels are under the tight control of multiple micro-RNAs (Zhou et al., 2016; Dai et al., 2020; Grad et al., 2022). With the notion that the *PTPRU* gene yields multiple protein isoforms, among which nuclear-localized ones (Liu et al., 2014), it is clear that there remain many riddles to solve.

### 2.17 *PTPRN* and *PTPRN2*

Also the last couple of classical PTP genes to be discussed, *PTPRN* and *PTPRN2*, encode transmembrane receptor-type PTPs that lack phosphotyrosine phosphatase activity: IA-2 and IA-2β. Their first hundred or so N-terminal amino acids represent a RESP18 domain that is homologous to glucocorticoid-responsive protein regulated endocrine-specific protein 18 (Figure 1) and that is responsible for their routing towards dense core secretory vesicles in cells (Sosa et al., 2016). *PTPRN* and *PTPRN2* are both expressed in neuro-endocrine cells and proteolytic processing of their products (Trajkovski et al., 2004) yields protein fragments that represent major autoantigens in type 1 diabetes and are critical regulators of endocrine secretion in adrenal, pancreatic and brain tissue (Cai and Notkins, 2016). The molecular mechanisms they can exploit to exert their tasks are manyfold. The proteins’ subcellular routing and step-wise cleavage enables them to sequentially help in assembling secretory vesicles, support membrane fusion and cargo release, and in the end translocate to the nucleus to boost transcription of appropriate target genes (Trajkovski et al., 2004; Cai and Notkins, 2016). Moreover, IA-2 and IA-2β are able to homo- and hetero-dimerize, also with other transmembrane PTPs and then downregulate the activity of these other RPTPs (Gross et al., 2002). There is a remarkable difference, though, between the two highly related genes; *PTPRN* spans some 20 kbp of DNA whereas *PTPRN2* covers no less than one Mbp of DNA. Furthermore, whereas IA-2 is still considered a phosphatase-dead molecule, IA-2β has meanwhile been unmasked as a phospholipid phosphatase (Caromile et al., 2010; Sengelaub et al., 2016).

Despite encoding a major type I diabetes autoantigen and given its impact on insulin secretion (Saeki et al., 2002), not a single *PTPRN* polymorphism has been unambiguously linked to neuroendocrine diseases. Likely, the compact size of the gene severely reduced the gene’s chance to gather sequence variants that result from replication errors. Nevertheless, a recent approach using a Kullback-Leibler-type statistical method did suggest gene-gene interactions involving *PTPRN* effects on type 2 diabetes status (Chen et al., 2019). The involved SNPs (rs10245268 and rs2335845), however, that are mentioned in the study do not reside in the *PTPRN* locus but rather represent *PTPRN2* intronic variants.

The sheer size of *PTPRN2* parallels the considerable amount of sequence variants that have been deposited for the gene, and for several of them disease associations have been reported. Following up on the diabetes link mentioned just above, the simple tandem repeat sequences (STRs) in *PTPRN2* are viewed as a source for somatic mutations that could trigger an autoimmune response as in type 1 diabetes (Ross, 2014). Furthermore, since *PTPRN2* hosts several other genes intragenically, it may be that some of those long-noncoding- and micro-RNA genes make part of a joint regulatory network in pancreas and brain with relevance for insulin and neurotransmitter release, as was recently suggested for microRNA miR-153 and its host gene (Mandemakers et al., 2013). This regulation may well involve DNA methylation, as *PTPRN2*’s methylation status has been associated with immunological (Li Yim et al., 2016; Zimmermann et al., 2016) and neurological disorders (Do et al., 2016). Expression levels for the gene indeed matter, given the reports that the detection of CNVs and RNA-seq data connect *PTPRN2* with neurodevelopmental disorders (Mosca et al., 2016) including autism (Filosi et al., 2020), attention deficit hyperactivity disorder (Lionel et al., 2011) and syndromes involving hearing impairments (Abu-Amero et al., 2013). Behavioral pathologies are also mirrored in IA-2β deficient mice (Nishimura et al., 2009), and this connection with hearing defects is corroborated by the identification of rs10081191 in *PTPRN2* being associated with noise-induced hearing loss in a Chinese cohort (Niu et al., 2021). Both epigenetic and genetic (rs1670344) determinants of *PTPRN2* expression were also linked to childhood obesity (Lee, 2019), which seems to fit the finding that an intronic SNP in *PTPRN2* (rs2091718; *p* = 7.2 × 10^–9^) was associated with sweet taste and sugary food preferences in an exploratory GWAS analysis (Fernandez-Carrion et al., 2021). Finally, an IA-2β p.I806V variant (rs1257461683) was identified through WES in one family with familial clustering of ischemic stroke (Ilinca et al., 2020), and an IA-2β frameshift mutant (p.Y805fs) ended up in ClinVar but without a phenotypic description (VCV000789293).

## 3 Discussion

Phosphorylation, not only of proteins, is an extremely powerful chemical way to steer biological processes in a fast and reversible way. Phosphorylation of tyrosine residues in effector proteins has evolved as a major signalling method to control almost every decision in multicellular organisms. Thus it makes sense to expect that protein tyrosine phosphatases (PTPs), being the counter-enzymes of protein tyrosine kinases in this mechanism, have a major role in the normal development and function of such organisms, including humans. As a consequence, inborn errors in PTP genes should be encountered when cataloguing the molecular mechanisms of diseases. The alternative, that all members of this huge family of genes (Alonso et al., 2004; Alonso et al., 2016; Alonso and Pulido, 2016) are so essential that polymorphisms affecting their structures and functions are incompatible with life, is very unlikely. After all, the evolutionary tree of the PTP family suggests ample opportunity for ambiguity and redundancy, and knock-out mouse models for many of the classical PTP genes do exist and reveal rather subtle phenotypes ((Hendriks et al., 2013) and references therein). Here, we focused on hereditary variants of the 37 classical PTP genes in human that can be extracted from databases and literature and that are suspect of a disease link. The resulting list is overwhelming but largely consists of associations and correlations. In the minority of cases a clear-cut genotype-phenotype correlation could be entered in the Online Mendelian Inheritance in Man database. Therefore, if our review serves a purpose we hope that this will be the following: a strong plea for i) genetic research aimed at clearly separating the disease-relevant from the benign variants using inclusive datasets, and ii) molecular analysis that documents the precise effects of gene variants, first perhaps the frame-shift, missense and nonsense variants and at a later stage the many genetic changes that likely impact on gene regulation, such as intronic and intergenic SNPs, indels, STR dynamics, CNVs and so on. To add to the complexity, also epigenetic information – i.e. non-DNA sequence-based information, such as DNA methylation, modification of histone proteins, and regulatory powers based on 3D genome organization – is likely to contribute to heritable phenotypes. In support, several disease associations have already been deduced on the basis of the PTP gene’s methylation status (Yeh et al., 2006; Chen et al., 2015; Do et al., 2016; Li Yim et al., 2016; Zimmermann et al., 2016; Lee, 2019).

### 3.1 Genetic investigations

It is to be expected that the continuing improvements on acquiring and analysing genomic data in the end will help us to move beyond the mere collection of sequences and bring us in the phase that we can use machine (and machine learning) to filter out the meaningful causal relations, also for multigenic complex traits, rare diseases and the full array of populations (Burgess, 2022). The same puzzle is presented for somatic changes in cancer specimens and although patterns are being discerned for the non-coding variants (Dietlein et al., 2022) it remains a tremendous challenge to separate the causal and bystander mutations. The situation becomes even more daunting when also the regulatory possibilities at the post-transcriptional level have to be taken into account. Alternative splicing is already a firmly confounding factor, but also the extensive network of micro-RNA mediated control of RNA stability and translation control adds a bewildering dimension to the problem. Multiple classical PTP genes harbour transcriptional units on the complementary strand that are annotated as antisense, microRNA, lncRNA or circRNA gene, and that are impacting, at least, on transcripts originating from their host gene (Mandemakers et al., 2013; Xing et al., 2014; Abuhatzira et al., 2015; Yao et al., 2015; Zhou et al., 2016; Han et al., 2019; John et al., 2019; Wei et al., 2019; Dai et al., 2020; Ma et al., 2020; Shi et al., 2020; Grad et al., 2022; Wu et al., 2022). Intriguingly, also in return PTPs may have bearing for microRNAs themselves, as was recently noted for PTP1B’s impact on the phosphorylation level of the argonaute two protein, and thus its association with miRNAs (Coulis et al., 2022). Obviously, all these levels of (reciprocal) interactions provide a real challenge to map genotype-phenotype effects for the overwhelming stream of gene variants now that the whole-genome sequencing locks have opened.

### 3.2 Molecular investigations

Indeed an enormous amount of work needs to be done and, as a reward, much knowledge is to be gained. Having bio-assays up and running and protein domain structures at hand it is nowadays straightforward, e.g. by exploiting CRISPR/Cas9 based geno-copying techniques, to experimentally check on effects by missense mutations in cell models and model organisms. However, we should realize that we still know relatively little of our proteins and thus should keep an open eye for moonlighting (Singh and Bhalla, 2020), i.e. additional processes that the PTP under study may be involved in. Furthermore, although structural biology has received powerful assistance from machine learning, even in this post-AlphaFold era there remain considerable gaps in our knowledge on protein structures (David et al., 2022). Notably transmembrane proteins and multiprotein complexes are largely off the radar, let alone the extensive post-translational modification landscape, including proteolytic scenarios, that adds a layer of complexity to the functional interpretation of apparently simple amino acid changes. Furthermore, a considerable part of the proteome will present so-called intrinsically disordered regions, protein segments that are involved in coacervation – a relatively new phenomenon in molecular cell biology. Coacervation is a reversible process of de-mixing a homogeneous solution of one or more (bio)molecules into two distinct phases; a dispersed phase and a condensed phase. The latter can be viewed as a kind of membrane-less organelle, which impinges on protein localization, activity and/or stability. The field is still in its infancy but the relevance of biomolecular coacervates for cellular processes, notably signalling, is evident (Su et al., 2021b) and, supported by the recent report of variant SHP1-R360E (Zhang et al., 2022), we expect the PTP family to contribute its fair share.

### 3.3 In conclusion

Let us not end with emphasizing this daunting task ahead; allow us to point to a recent inspiring study in which a high-throughput approach worked well to graze a landscape with hundreds of autoimmune disease-associated loci and enabled the definition of five dozen putatively causal variants, subsequently one of these SNPs was introduced in mice and human cells to allow disclosure of its physiologically relevant effect (Mouri et al., 2022). Thus, although thousands of verdicts are still out, in the end we will be able to prove hereditable variants of classical PTP genes either innocent or guilty.
